# Nwd1 Regulates Neuronal Differentiation and Migration through Purinosome Formation in the Developing Cerebral Cortex

**DOI:** 10.1016/j.isci.2020.101058

**Published:** 2020-04-13

**Authors:** Seiya Yamada, Ayaka Sato, Shin-ichi Sakakibara

**Affiliations:** 1 Laboratory for Molecular Neurobiology, Graduate School of Human Sciences, Waseda University, 2-579-15 Mikajima, Tokorozawa, Saitama 359-1192, Japan

**Keywords:** Developmental Neuroscience, Cellular Neuroscience, Stem Cells Research

## Abstract

Engagement of neural stem/progenitor cells (NSPCs) into proper neuronal differentiation requires the spatiotemporally regulated generation of metabolites. Purines are essential building blocks for many signaling molecules. Enzymes that catalyze *de novo* purine synthesis are assembled as a huge multienzyme complex called “purinosome.” However, there is no evidence of the formation or physiological function of the purinosome in the brain. Here, we showed that a signal transduction ATPases with numerous domains (STAND) protein, NACHT and WD repeat domain-containing 1 (Nwd1), interacted with Paics, a purine-synthesizing enzyme, to regulate purinosome assembly in NSPCs. Altered Nwd1 expression affected purinosome formation and induced the mitotic exit and premature differentiation of NSPCs, repressing neuronal migration and periventricular heterotopia. Overexpression/knockdown of Paics or Fgams, other purinosome enzymes, in the developing brain resulted in a phenocopy of Nwd1 defects. These findings indicate that strict regulation of purinosome assembly/disassembly is crucial for maintaining NSPCs and corticogenesis.

## Highlights

• STAND protein Nwd1 interacts with Paics to regulate the purinosome formation
• Dysregulated expression of Nwd1 induced the premature differentiation of NSPCs
• Nwd1 KD repressed the neuronal migration, causing the periventricular heterotopia
• Tightly regulated assembly of purinosome components is crucial for corticogenesis

## Graphical Abstract

**Figure:**
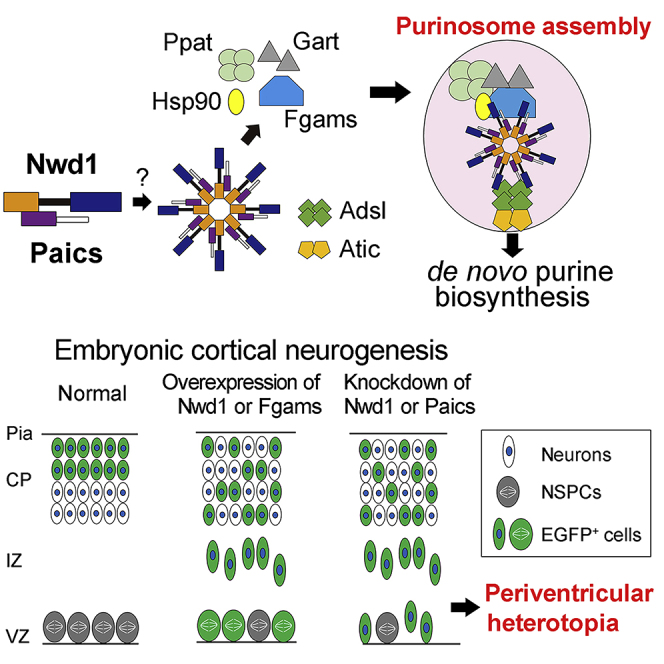

## Introduction

The spatiotemporal differentiation of neural stem/progenitor cells (NSPCs) into immature neurons and neuronal migration are necessary for the proper development of the central nervous system (CNS). The cerebral cortex of embryonic mice contains two distinct types of NSPCs: paired box 6-positive (Pax6^+^) apical progenitor cells (radial glia), located in the ventricular zone (VZ), and T-box brain protein 2-positive (Tbr2^+^) basal progenitor cells (intermediate progenitor cells), which are located in the subventricular zone (SVZ) (Englund et al., 2005). In the neocortex, newborn neurons generated from NSPCs migrate radially toward the cortical plate, accompanied by sequential changes in cell shape. Neurite outgrowth and ensuing polarity formation in immature neurons are also required for cortical layer stratification, and defects in neuronal migration not only cause brain malformation but also various psychiatric disorders, including epilepsy and mental retardation (Hansen et al., 2017, Represa, 2019).

Purines, compounds containing a pyrimidine ring fused with an imidazole ring, are found in all living species and include the nucleobases adenine and guanine (Traut, 1994). Apart from their critical function as the building blocks of DNA (deoxyadenosine and deoxyguanosine) and RNA (adenosine and guanosine), purines work as components of essential biomolecules and as a source of second messenger molecules (cyclic AMP and cyclic GMP), cofactors coenzyme A and nicotinamide adenine dinucleotide (NADH), cellular energy substrate ATP, and GTP, which is essential for the signal transduction of a large number of G-proteins. Other purine derivatives contain hypoxanthine, xanthine, and uric acid. Specifically, purines function as neurotransmitters in the brain by acting upon purinergic receptors. Purine metabolites, including ATP and GTP/GDP, are crucial for polarity formation in postmitotic cortical neurons (Raman et al., 2018). During brain development, purinergic signaling is essential for NSPC maintenance and neuronal migration in the neocortical SVZ (Lin et al., 2007, Liu et al., 2008).

In mammalian cells, purine content is regulated by a coordinated balance between the *de novo* and salvage biosynthetic pathways. Although the cellular purine pool is usually supplied by the recycling of degraded bases via the salvage pathway, the *de novo* pathway is upregulated under cellular conditions demanding higher levels of purines and their derivative nucleotides, such as tumor growth and cell proliferation (Yamaoka et al., 1997). *De novo* purine synthesis comprises a series of 10 enzymatic reactions and is mediated by six evolutionarily conserved enzymes (phosphoribosyl pyrophosphate amidotransferase [PPAT], phosphoribosylglycinamide formyltransferase [GART], formylglycin-amidine ribonucleotide synthase [FGAMS], phosphoribosylaminoimidazole carboxylase phosphoribosylaminoimidazole succinocarboxamide synthetase [PAICS], adenylosuccinate lyase [ADSL], and 5-aminoimidazole-4-carboxamide ribonucleotide formyltransferase inosine monophosphate [IMP] cyclohydrolase [ATIC]), to produce IMP from phosphoribosylpyrophosphate (Baresova et al., 2018). The enzymes that catalyze *de novo* purine synthesis are assembled near mitochondria and microtubules as a huge multienzyme complex called “purinosome” (An et al., 2008, An et al., 2010, French et al., 2016). Purinosome is a dynamic and functional giant protein complex that emerges during high levels of cellular purine demand in mammalian cultured cells (An et al., 2008). Purinosome formation is linked to cell division (Chan et al., 2015). Furthermore, the dynamic assembly and disassembly of purinosomes *in vivo* might be crucial for the proper development of the human brain. Mutations in *ADSL* and *ATIC* genes cause severe developmental brain defects, such as mental retardation, autistic features, epilepsy, microcephaly, and congenital blindness (Jurecka et al., 2015, Marie et al., 2004). The bifunctional enzyme PAICS, another component of the purinosome, is associated with prostate and breast cancer metastasis and proliferation (Barrfeld et al., 2015, Chakravarthi et al., 2018, Meng et al., 2018). PAICS deficiency in humans was recently reported. A missense mutation in *Paics* causes the severe phenotype with multiple malformations, including a small body, short neck, and craniofacial dysmorphism, resulting in early neonatal death (Pelet et al., 2019). To date, however, there is no direct evidence of the localization or physiological function of purinosomes during brain development.

It is known that the adult brain preferentially uses the purine salvage synthetic pathway over the *de novo* pathway. Terminally differentiated neurons require large amounts of ATP, which is mainly derived from the purine salvage pathway and produced in mitochondria. Genetic defects in the salvage pathway cause nucleotide imbalance, leading to their depletion in the mitochondria and severe neurological diseases including Lesch-Nyhan syndrome and mitochondrial DNA depletion syndrome (Fasullo and Endres, 2015). It is highly likely that a tightly controlled balance between the *de novo* purine pathway and the purine salvage pathway is necessary for healthy brain development. However, the molecular mechanism that determines this balance remains obscure.

Previously, we identified the NACHT and WD repeat domain-containing protein 1 (*Nwd1*) gene and showed that the Nwd1 protein is expressed in NSPCs and immature neurons in the cerebral cortex of embryonic mice (Yamada and Sakakibara, 2018). The Nwd1 protein contains a NACHT domain, which is predicted to have nucleoside-triphosphatase (NTPase) activity, in the central region and a cluster of WD40 repeats at the C terminus. Based on the domain structure, Nwd1 is designated as a member of signal transduction ATPases with numerous domains (STAND) protein superfamily and is conserved across species, including zebrafish, mice, rats, monkeys, and humans (Yamada and Sakakibara, 2018). Other members of the STAND protein family often mediate ligand-induced self-oligomerization to form the giant multiprotein complex critical for various important cellular responses; e.g., the apoptotic peptidase activating factor 1 (Apaf1) and nucleotide-binding oligomerization domain-like receptors (NLRs) induce the assembly of large multiprotein complexes, the “apoptosome” and “inflammasome,” and play central roles in cell death and innate immune responses, respectively (Cai et al., 2017, Dorstyn et al., 2018, Leipe et al., 2004). Although the cellular function of Nwd1 remains unclear, its domain structure is analogous to Apaf1, an essential molecule for apoptosome assembly, which is required for apoptosis initiation (Dorstyn et al., 2018, Yamada and Sakakibara, 2018). Our current study shows that Nwd1 regulates NSPC proliferation and neuronal migration through the control of purinosome formation during cortical development. These findings would shed light on a machinery governing the purine metabolism in nervous system.

## Results

### Nwd1 Overexpression In Vivo Increases the NSPCs Fraction and Delays the Radial Migration of Immature Neuron

**Figure 1:**
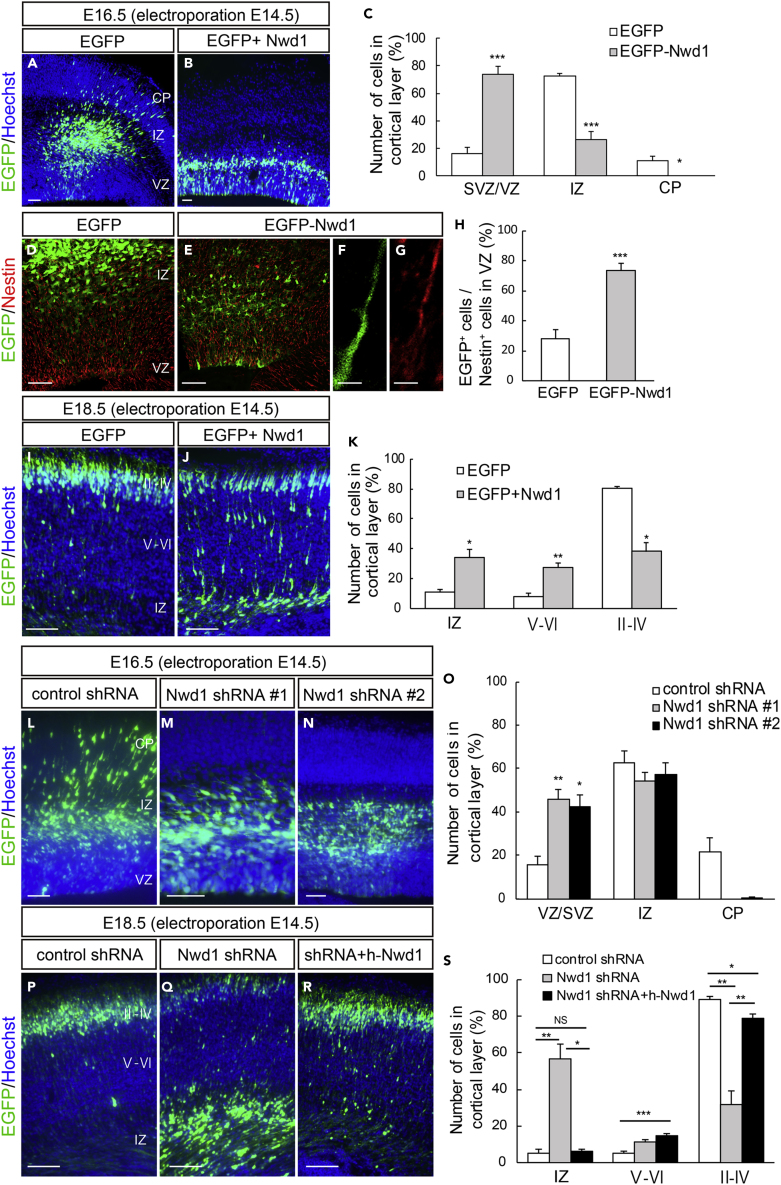
Dysregulation of Nwd1 Disturbs the Radial Migration of Neurons and Directs NSPCs to Reside in the VZ/SVZ (A–K) Nwd1 overexpression in the embryonic neocortex. (A–C) *In utero* electroporation of control EGFP (A) or Nwd1 together with EGFP (B) was performed on E14.5, and the neocortex was analyzed at E16.5. (C) Distribution of electroporated EGFP^+^ cells in the indicated areas. ∗p < 0.05, ∗∗∗p < 0.001, Welch's t test followed by Holm-Bonferroni correction. (D–H) EGFP-Nwd1 (E) or control EGFP (D) was electroporated at E14.5, and brains were immunostained for Nestin (red) at E16.5. (F and G) Higher magnification of the VZ cells expressing EGFP-Nwd1 (F) and Nestin (G). (H) Ratio of EGFP^+^ or EGFP-Nwd1^+^ cells to the total number of Nestin^+^ cells in the VZ. ∗∗∗p < 0.001, Welch's t test. (I–K) Nwd1 or control EGFP was electroporated at E14.5, and the brains were collected on E18.5. (K) Distribution of EGFP^+^ cells in the indicated layers. ∗p < 0.05, ∗∗p < 0.01, Welch's *t* test followed by Holm-Bonferroni correction. (L–S) Nwd1 knockdown represses neuronal migration. An *Nwd1* shRNA (shRNA #1 or shRNA #2) was co-electroporated with EGFP at E14.5 and cortices were analyzed at E16.5 (L–O) or E18.5 (P–S). (O) Distribution of EGFP^+^ cells in the indicated areas at E16.5. ∗p < 0.05, ∗∗p < 0.01, Welch's t test followed by Holm-Bonferroni correction. (R) *Nwd1* shRNA was co-electroporated with the full-length human *NWD1* cDNA at E14.5, and the cortex was analyzed at E18.5. (S) Distribution of EGFP^+^ cells in the indicated areas at E18.5. NS, not significant; ∗p < 0.05, ∗∗p < 0.01, ∗∗∗p < 0.001, Welch's t test followed by Holm-Bonferroni correction. All data are presented as means ± SEM. Scale bars, 50 μm in (A), (B), (D), €, and (L)–(N); 5 μm in (F) and (G); 100 μm in (I), (J), and (P)–(R).

To investigate the role of Nwd1 in the developing cerebral cortex, we overexpressed the *Nwd1* gene *in vivo* using *in utero* electroporation. Full-length Nwd1 or control EGFP was electroporated into NSPCs in the developing dorsal neocortex at E14.5, a stage at which extensive neurogenesis and neuronal migration occurs. Electroporated embryos were harvested and analyzed after 48 h (at E16.5). To visualize the electroporated cells, the EGFP reporter plasmid was co-electroporated with the *Nwd1* plasmid into the same embryos. Figures 1A–1C show that Nwd1 overexpression significantly suppressed neuronal migration from VZ, causing the accumulation of Nwd1-overexpressing cells in VZ/SVZ (control, 16.5 ± 4.2%, n = 6; Nwd1, 73.7 ± 6.0%, n = 6). At E16.5, the majority of cells electroporated with the control EGFP plasmid had migrated and reached the intermediate zone (IZ) and cortical plate (CP), where they became positive for Tbr1, a marker for post-mitotic neurons in the deep cortical layers and subplate (IZ, 72.3 ± 2.5%; CP, 11.2 ± 3.3%) (Figures 1A and S2A–S2C). However, Nwd1-overexpressing cells were rarely observed within the CP (Figures 1B, 1C, and S2D–S2F). Many Nwd1^+^ cells remaining within the VZ/SVZ were positive for the neural stem cell marker Nestin (Figures 1D–1H) (control, 29.0 ± 6.0%, n = 4; EGFP-Nwd1, 73.8 ± 4.8%, n = 4), suggesting that they retained their NSPC nature and lined the ventricular wall for at least 2 days, without moving toward the pial surface. After 4 days (at E18.5), EGFP expression was observed in the Brn2^+^ upper cortical layers (II–IV) and was almost absent in the Brn2^-^ deep cortical layers (V and VI) or IZ in controls (Figures 1I and S2J–S2L). At this time, cells overexpressing Nwd1 remained in the lower layers of the neocortex, including IZ and SVZ (Figures 1J and 1K) (layers II–IV: control, 80.5 ± 1.2%, n = 4 versus Nwd1, 38.4 ± 6.1%, n = 4; layers V–VI: control, 8.3 ± 1.9% vs. Nwd1, 27.6 ± 2.7%; IZ: control, 11.3 ± 1.8% vs. Nwd1, 34.0 ± 5.7%). Within the Brn2^-^ lower cortical layers, Nwd1-overexpressing cells exhibited the elongated bipolar morphology of traveling immature neurons (Figures 1J and S2M–S2O). These observations indicated that Nwd1 overexpression causes a significant increase in the Nestin^+^ NSPC pool accumulating in the VZ/SVZ and delays the radial migration of immature neurons.

### Nwd1 Knockdown Causes Premature Differentiation of NSPCs and Represses Neuronal Migration

We explored the effect of Nwd1 loss of function on cortical development using small hairpin RNA (shRNA) delivery *in vivo* via *in utero* electroporation. Two different shRNA constructs (shRNA #1 and shRNA #2) targeting the coding region of mouse *Nwd1* significantly reduced Nwd1 protein expression levels (Figure S1A). shRNA specificity was further demonstrated by Nwd1 immunostaining. Endogenous Nwd1 protein expression in cultured NSPCs was silenced by shRNA constructs (Figures S1B–S1E). We co-electroporated one of the shRNA constructs with an EGFP-expression plasmid into the neocortex at E14.5 and harvested embryos after 2 or 4 days. Then, we assessed the distribution of EGFP^+^ cells among the discrete cortical zones. In control embryos, almost all EGFP^+^ cells were found in either IZ or CP, and only a small fraction of cells was observed in VZ/SVZ at E16.5 (Figure 1L). However, *Nwd1* knockdown (KD) resulted in a drastically reduced cell migration into the Tbr1^+^ CP as a large number of cells remained in VZ/SVZ at E16.5 (% of cells in the VZ/SVZ: control, 15.7 ± 3.7%, n = 8; *Nwd1* shRNA #1, 45.9 ± 4.2%, n = 4; *Nwd1* shRNA #2, 42.2 ± 5.7%, n = 5) (Figures 1L–1O and S2G–S2I). We noticed that *Nwd1* KD cells accumulated in IZ if they were unable to penetrate the boundary between IZ and CP (Figures 1M and 1N). This phenotype was more pronounced after a further 2 days of shRNA expression. At E18.5, *Nwd1* KD caused a significant accumulation of cells in IZ (control, 5.4 ± 2.0%, n = 4; *Nwd1* shRNA #1, 56.8 ± 7.8%, n = 5) (Figures 1P–1S). Consequently, fewer *Nwd1* KD cells reached the upper cortical layers (layers II–IV, control, 89.4 ± 1.3%, n = 4; *Nwd1* shRNA #1, 32.0 ± 7.1%, n = 5; layers V–VI: control, 5.2 ± 1.0%; *Nwd1* shRNA #1, 11.3 ± 1.4%). Many *Nwd1* KD cells were still observed as Brn2^+^ cells in the deep cortical layers (Figures 1P–1S and S2Q). These defects were rescued by overexpression of the human NWD1 homolog, which is resistant to targeting by the mouse *Nwd1* shRNA. We co-electroporated *Nwd1* shRNA and the full-length human *NWD1* cDNA into the E14.5 cerebral cortex and performed analysis at E18.5. A large fraction of the electroporated cells reached the upper cortical layers through IZ (Figures 1Q and 1S) (% of cells in layers II–IV, 79.0 ± 2.4%; layers V–VI, 14.8 ± 1.3%; IZ, 6.3 ± 1.6%, n = 10), restoring the cellular distribution comparable with that of the non-targeting control (see above). This finding further supported the notion that the loss of function of *Nwd1* causes a severe migratory defect in immature neurons *in vivo*.

**Figure 2:**
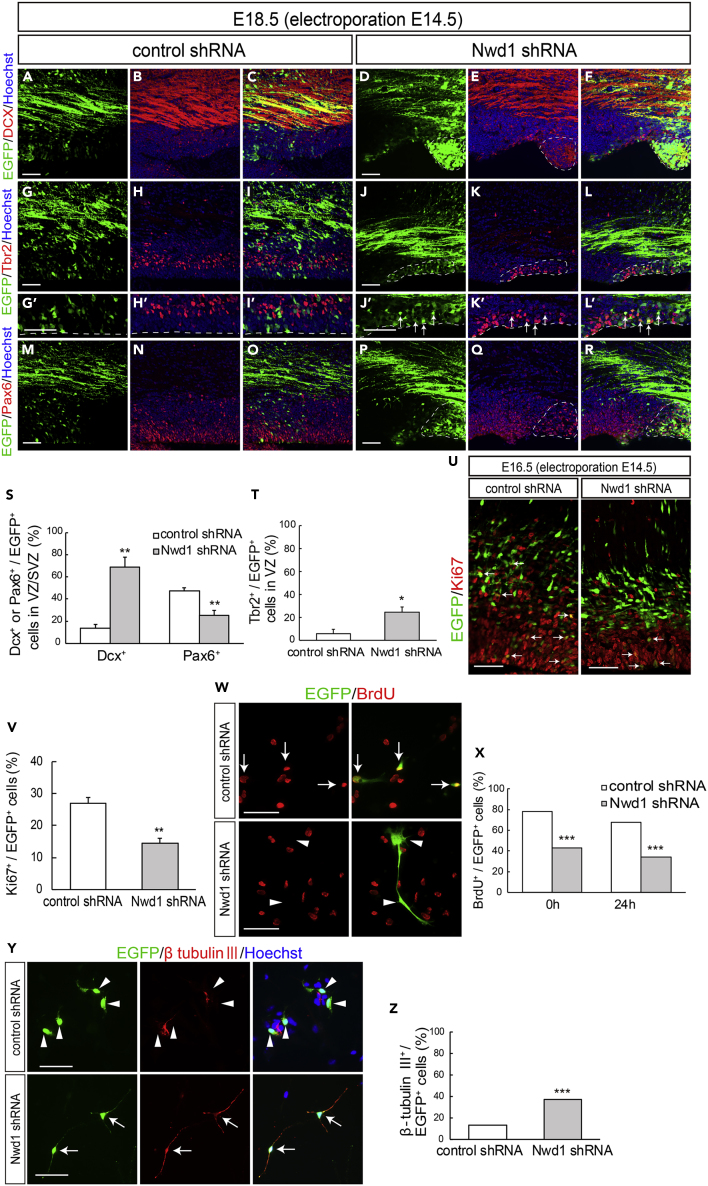
Nwd1 Knockdown Causes the Cell-Cycle Exit and Premature Differentiation of NSPCs (A–T) A control or *Nwd1* shRNA was electroporated together with EGFP at E14.5, and the cortices were harvested at E18.5. Confocal images of sections stained with anti-Dcx (A–F), anti-Tbr2 (G–L′), and anti-Pax6 (M–R). (G′–L′) Higher magnification of the VZ cells shown in (G–L). The areas surrounded by dashed lines denote the distribution of cells that were electroporated with the *Nwd1* shRNA (green), which remained in ZV/SVZ areas at E18.5. The VZ surface is outlined by the dashed line. (S) Quantification of EGFP^+^ Dcx^+^ or EGFP^+^ Pax6^+^ cells to total EGFP^+^ cells in VZ/SVZ. Data are presented as means ± SEM. ∗∗p < 0.01, Welch's t test. (T) Quantification of EGFP^+^ Tbr2^+^ cells to total EGFP^+^ cells in VZ. Data are presented as means ± SEM. ∗p < 0.05, Welch's t test. (U and V) An *Nwd1* shRNA (U, right panel) or control shRNA (U, left panel) was co-electroporated with EGFP at E14.5, and the cortices were stained for the Ki67 mitotic marker (red) at E16.5. Arrows indicate EGFP^+^ Ki67^+^ cells. (V) Quantification of EGFP^+^ Ki67^+^ cells in relation to the total number of EGFP^+^ cells. Data are presented as means ± SEM. ∗∗p < 0.01, Welch's t test. (W and X) Primary cultured NSPCs were electroporated with control shRNA (W, upper panels) or Nwd1 shRNA (W, lower panels) together with EGFP, followed labeling with BrdU (red) for 24 h. Arrows indicate EGFP^+^ BrdU^+^ proliferating NSPCs. Arrowheads indicate EGFP^+^ BrdU^–^ cells. (X) Following BrdU administration, NSPCs were fixed at 0 or 24 h and the BrdU^+^ cells were counted. The numbers of EGFP^+^ BrdU^+^ cells were compared using the chi-square test. ∗∗∗p < 0.001, 0 h: control shRNA, n = 240; Nwd1 shRNA, n = 153; 24 h: control shRNA, n = 214; Nwd1 shRNA, n = 111. (Y and Z) Primary cultured NSPCs, electroporated with control shRNA (Y, upper panels) or Nwd1 shRNA (Y, lower panels) along with EGFP, were treated for 24 h with differentiation induction medium containing 1% FBS, followed by immunostaining with β-tubulin III antibody (red). Nuclei were stained with Hoechst dye (blue). Arrows indicate EGFP^+^ β-tubulin III ^+^ neurons. Arrowheads indicate EGFP^+^ β-tubulin III ^–^ cells. (Z) The numbers of EGFP^+^ β-tubulin III^+^ neurons were compared using the chi-square test. ∗∗∗p < 0.001, control shRNA, n = 503; Nwd1 shRNA, n = 240. Scale bars, 50 μm.

We previously reported the substantial expression levels of Nwd1 in the VZ/SVZ and immature neurons (Yamada and Sakakibara, 2018). Accordingly, a larger number of cells overexpressing Nwd1 remained within VZ/SVZ (Figure 1). Thus, we examined whether *Nwd1* KD affected the nature of the NSPC pool in VZ/SVZ. At E18.5, i.e., 4 days after shRNA electroporation, double immunostaining revealed that the *Nwd1* KD cells remaining in VZ/SVZ were positive for doublecortin (Dcx) (control, 13.6 ± 3.7%, n = 7; Nwd1, 68.7 ± 9.2%, n = 5) (Figures 2A–2F and 2S) and β tubulin III (Figures S3A–S3D), which are markers of newborn immature neurons. Interestingly, *Nwd1* KD drove many VZ cells to ectopically and prematurely express Tbr2, a marker of the SVZ basal progenitor cells (intermediate progenitor cells) (control, 6.0 ± 3.9%, n = 4; Nwd1, 24.7 ± 4.7%, n = 4) (Figures 2G–2L′ and 2T). Concurrently, we observed a decreased density of Pax6^+^ apical progenitors in the VZ region, where *Nwd1* shRNA was expressed (control, 47.2 ± 3.0%, n = 5; Nwd1, 25.6 ± 4.2%, n = 6) (Figures 2M–2S). Immunostaining for the mitotic marker Ki67 revealed that the proliferation rate of NSPCs was significantly reduced by *Nwd1* KD (control, 27.1 ± 1.7%, n = 6; shRNA #1, 14.4 ± 2.3%, n = 6) (Figures 2U and 2V). Terminal deoxynucleotidyl transferase dUTP nick-end labeling (TUNEL) staining of E16.5 brains revealed that *Nwd1* KD did not induce apoptosis (control shRNA, 0.7 ± 0.3%, n = 8; Nwd1 shRNA, 1.2 ± 0.3%, n = 8) (Figures S3E–S3I). To directly assess the effect of Nwd1 KD on NSPC proliferation, Nwd1 shRNA or control shRNA was transferred into primary cultured NSPCs then labeled with bromodeoxyuridine (BrdU). Following *in vitro* BrdU administration for 24 h, NSPCs were fixed after 0 or 24 h and the BrdU^+^ cells were counted. Nwd1 KD significantly decreased the number of BrdU-incorporated NSPCs (labeling index at 0 h: approximately 45%; 24 h: approximately 35%) relative to the control (0 h: approximately 80%; 24 h: approximately 70%) (Figures 2W and 2X). Conversely, the silencing of Nwd1 in NSPCs resulted in an accelerated commitment to the neuronal lineage. A differentiation assay of NSPCs indicated that Nwd1 KD increased the fraction of β tubulin III^+^ neurons (Figures 2Y and 2Z). Based on the early onset of lineage markers and decreased cell division rate *in vivo* and *in vitro*, we concluded that the loss of function of *Nwd1* induced the cell-cycle exit and premature neuronal differentiation of NSPCs. Abnormally produced progenies might follow neuronal differentiation near their place of birth, without proper cell migration, leading the apparent accumulation of EGFP^+^ cells within the VZ/SVZ in addition to the IZ (Figures 1O and 2F).

### Nwd1 Knockdown Causes Periventricular Nodular Heterotopia

**Figure 3:**
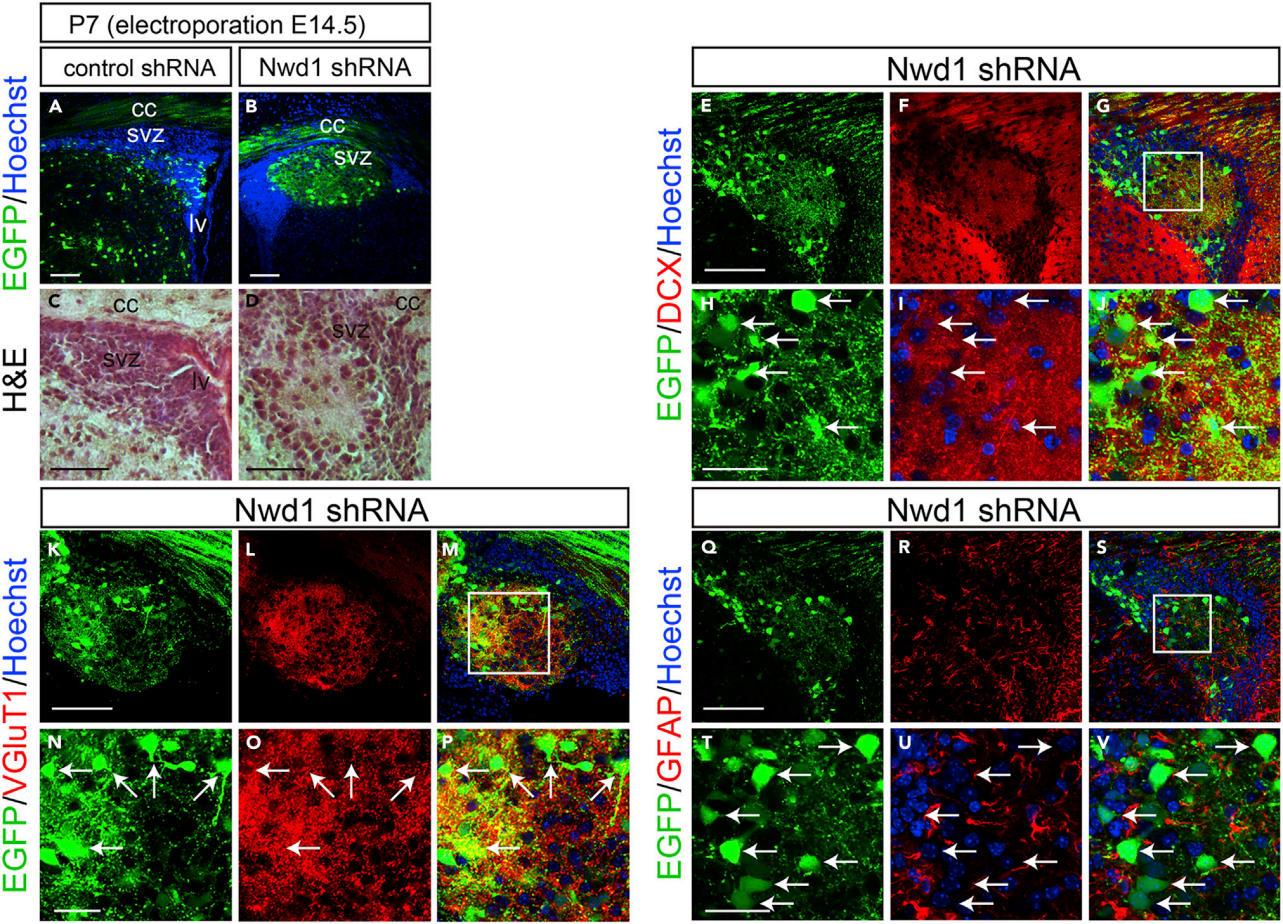
Nwd1 Knockdown Causes PH (A–V) A control or *Nwd1* shRNA was delivered into the brain together with EGFP on E14.5, and brains were collected on P7 (n = 5). (A and B) Sections through the SVZ area showing the development of periventricular heterotopia (HP) caused by *Nwd1* KD (B). Nuclei are counterstained with Hoechst dye (blue). (C and D) Hematoxylin and eosin staining of HP. HP regions were immunostained using anti-Dcx (E–J), anti-VGluT1 (K–P), or anti-GFAP (Q–V) antibodies. (H–J), (N–P), and (T–V) are higher magnifications of the boxed areas shown in (G), (M), and (S), respectively. Arrows indicate the abnormally differentiated glutamatergic neurons that were Dcx^+^, VGlut1^+^, and GFAP^–^. cc, corpus callosum; lv, lateral ventricle. Scale bars, 100 μm in (A)–(G), (K)–(M), and (Q)–(S); 30 μm in (H)–(J), (N)–(P), and (T)–(V).

We examined postnatal brains after the embryonic KD of *Nwd1*. Embryos electroporated with control shRNA or *Nwd1* shRNA at E14.5 were harvested at postnatal day 7 (P7), when neocortex stratification is almost complete. In a control experiment using a non-targeting shRNA, the electroporated cells were sparsely distributed in the entire cortical region, including the subcortical SVZ (Figure 3A). In contrast, *Nwd1* KD pups frequently developed “periventricular heterotopia,” manifested by ectopic nodular masses in the lining of the ventricular wall (Figures 3B and S4). These heterotopias were characterized by a lower cell density than the neighboring SVZ region and brighter nuclei, evidenced by Hoechst and hematoxylin staining (Figures 3A–3D). Most heterotopia-forming cells had large round cell bodies with few fine processes (Figures 3E and 3H), resembling neurons. Indeed, double immunostaining revealed that they were Dcx^+^ neuron (Figures 3E–3J). Notably, these neurons expressed the vesicular glutamate transporter 1 (VGlutT1), and their somata were also closely surrounded by multiple VGlutT1^+^ presynaptic terminals (Figures 3K–3P), implying the formation of excitatory circuits of glutamatergic neurons within a heterotopia. Conversely, these cells never exhibited labeling of the astrocyte marker GFAP (Figures 3Q–3V). Cellular architecture of this malformation was similar to the human periventricular nodular heterotopia composed of hyperexcitable neurons, which is a developmental cortical dysgenesis frequently characterized by focal drug-resistant epilepsy (Battaglia et al., 2006).

### Expression Levels of Nwd1 Is Crucial for Neurite Outgrowth and Axon Formation of Cortical Neurons

**Figure 4:**
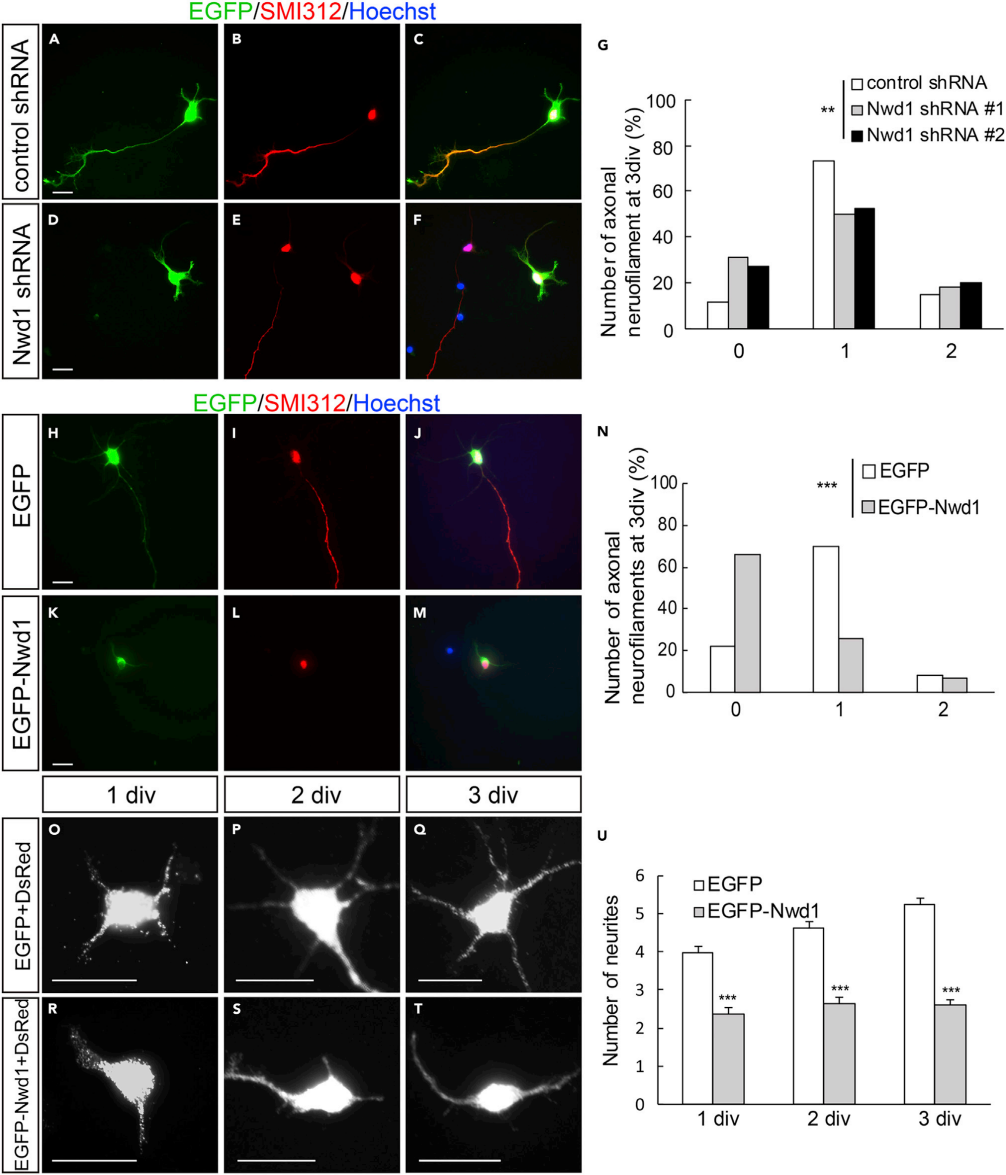
Tightly Regulated Nwd1 Expression Is Required for the Induction of Neuronal Identity (A–G) A non-targeting shRNA (A–C) or *Nwd1* shRNAs (D–F) were electroporated together with EGFP into primary cultured cortical neurons. Neurofilaments were stained with an anti-SMI312 antibody (red) at 3 div. Nuclei were stained with Hoechst dye (blue). (G) Number of SMI312^+^ axons extending from a single neuron. ∗∗p < 0.01 (chi-square test); control shRNA, n = 101; shRNA #1, n = 108; shRNA #2, n = 99. (H–N) Cortical neurons transfected with EGFP-Nwd1 (K–M) or control EGFP (H–J) were stained for SMI312 (red) at 3 div. Nuclei (blue) (N) Number of SMI312^+^ axons extending from a single neuron. ∗∗∗p < 0.001 (chi-square test); EGFP, n = 149; EGFP-Nwd1, n = 149. (O–U) EGFP-Nwd1 or control EGFP were electroporated into cortical neurons and cultured for 1 div (O and R), 2 div (P, S), and 3 div (Q and T). To visualize fine immature neurites, a dsRed expression plasmid was co-electroporated into the cells. (U) Number of neurites extending from a single neuron. Data are presented as means ± SEM. ∗∗∗p < 0.01, Welch's t test; EGFP 1 div, n = 150; 2 div, n = 150; 3 div, n = 200; EGFP-Nwd1 1 div, n = 150; 2 div, n = 150; 3 div, n = 200. Scale bars, 20 μm.

To understand Nwd1 cellular function in postmitotic differentiating neurons, loss-of-function and gain-of-function experiments were performed using primary cultured cortical neurons (Figure S5A). *Nwd1* shRNA constructs were transferred into dissociated neurons prepared from E16.5 embryos and cultured for 3 days *in vitro* (div). In the control, a large fraction (∼75%) of cells extended a single long axon immunostained for the SMI312 neurofilament marker (Figures 4A–4C). At this time, neurons transfected with *Nwd1* shRNA #1 and shRNA #2 exhibited fewer SMI312^+^ axons (Figures 4D–4F), and a notable number of cells lacked axons (Figure 4G). We assessed whether Nwd1 overexpression affected axonal extension in each cortical neuron. Control cells electroporated with EGFP usually had a single axon after culture for 3 div (Figures 4H–4J). In contrast, EGFP-Nwd1 expression inhibited axonal extension (Figures 4K–4M). These cells occasionally had few short neurites that were devoid of SMI312 immunoreactivity. To visualize all immature neurites extending directly from soma, a plasmid encoding the red fluorescent protein dsRed was co-electroporated into the cortical neurons and the total number of neural processes was counted as neurites. We found that EGFP-Nwd1 overexpression reduced the number of neurites by almost half at 1, 2, and 3 div (Figures 4O–4U) (1 div: control, 4.0 ± 0.2; EGFP-Nwd1, 2.4 ± 0.2; 2 div: control, 4.6 ± 0.2; EGFP-Nwd1, 2.6 ± 0.2; 3 div: control, 5.3 ± 0.1; EGFP-Nwd1, 2.6 ± 0.2). Compared with the control, each neurite appeared thinner and unbranched, suggesting early stage of neurite development. Consistently, in the embryonic cortex electroporated with *Nwd1* shRNAs *in utero*, we observed a significant number of apolar cells within the IZ, which had round cell bodies, and it appeared as if they had failed to transform into migratory spindle-shaped neurons (*arrows* in Figure S3L). These results indicated that *Nwd1* plays a vital role in axon formation in newborn neurons and that the highly controlled and just sufficient level of Nwd1 may be essential for axon and neurite outgrowth. Recent studies have indicated that dynamic changes in cell shape is closely coupled with the neuronal migration and cortical layer formation (Hirota and Nakajima, 2017). In the developing mammalian neocortex, newborn neurons transiently become multipolar cells with multiple neurites inside SVZ and lower IZ; thereafter, they undergo a change in morphology to a bipolar state before the onset of radial migration to the CP (Ohtaka-Maruyama and Okado, 2015); however, its molecular mechanism remains unclear. The defects in neuronal migration caused by manipulating *Nwd1* might reflect a principal function of this gene in the morphological transformation of neurons during neurogenesis.

### Nwd1 Protein Interacts with Paics

**Figure 5:**
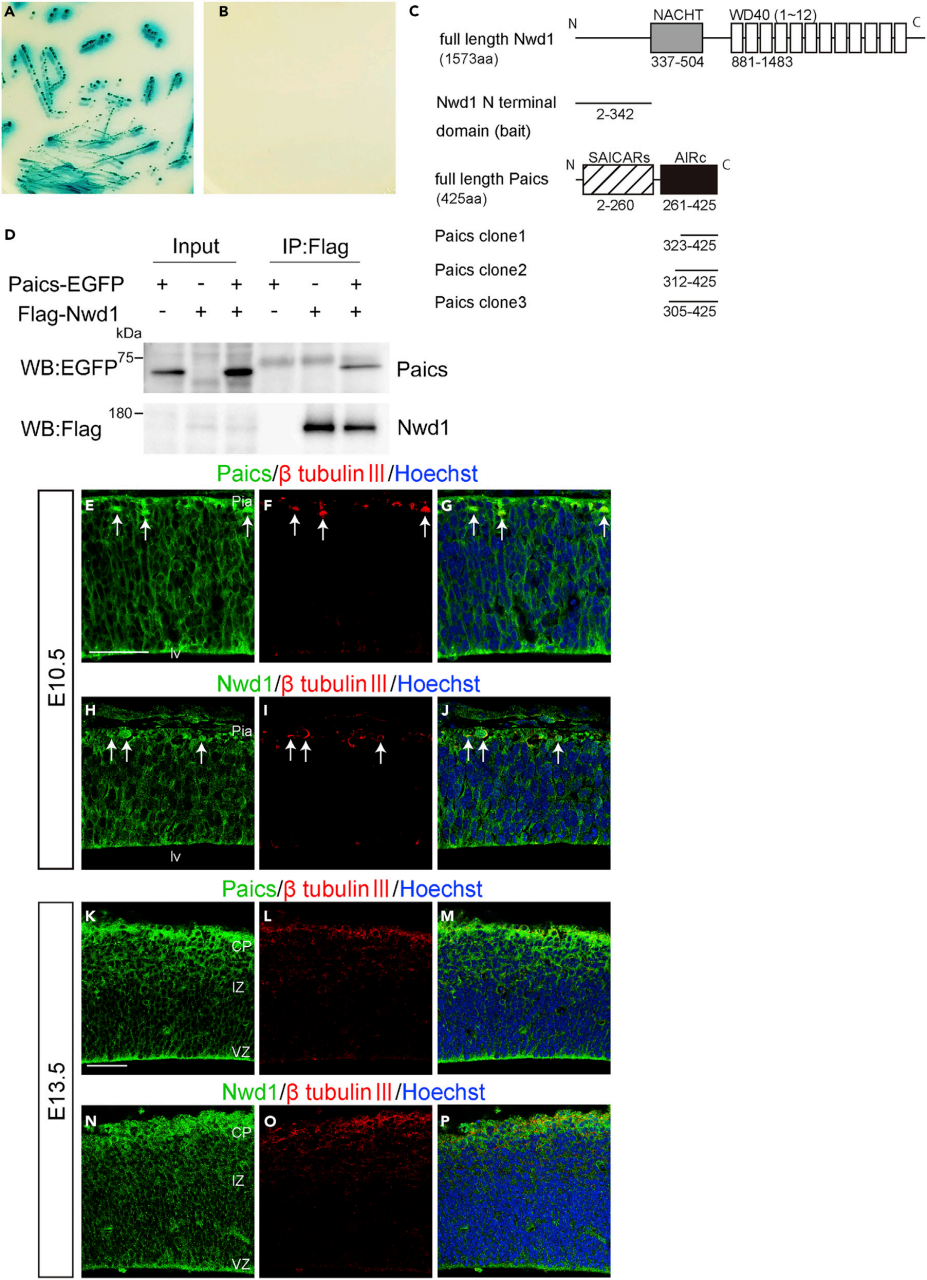
Nwd1 Interacts with Paics (A and B) A yeast transformant with pGBKT7-Nwd1 (bait) and pGADT7-Paics (prey) was streaked on SD agar plates containing quadruple dropout media with Aureobasidin A and X-α-Gal, showing a positive interaction (A, blue colonies). The absence of colonies indicated the negative control (B). (C) Domain structure of the mouse Nwd1 and Paics proteins. The Nwd1 N-terminal region was used as a bait, and the isolated *Paics* cDNAs are indicated. Gray box, NACHT domain; open boxes, WD40 repeats; hatched box, SAICARs activity domain; black box, AIRc activity domain. (D) Co-IP showing the interaction between Nwd1 and Paics. HEK293 cells expressing FLAG-Nwd1 and/or Paics-EGFP were subjected to immunoprecipitation with an anti-FLAG antibody, followed by immunoblotting with anti-EGFP or anti-FLAG antibodies. (E–P) Telencephalon at E10.5 (E–J) and E13.5 (K–P) were double immunostained with anti-Paics (E, K) or anti-Nwd1 (H, N) and anti-β-tubulin III (F, I, L, O) antibodies. Arrows indicate β-tubulin III ^+^ neurons. Pia, Pial surface; lv, lateral ventricle. Scale bars, 50 μm in (E)–(P).

We attempted to understand the molecular mechanism by which Nwd1 regulates cortical development. We used a Y2H screen to identify proteins interacting with Nwd1. Based on its structural similarity to other STAND-family proteins (Leipe et al., 2004), we hypothesized that the N-terminal region of Nwd1 serves as an effector domain by which the protein binds signaling molecule(s) to trigger self-oligomerization mediated by the NACHT domain and WD40 repeats. The N-terminal region of Nwd1 contains a DUF4062 motif, a functionally uncharacterized motif found in bacteria and eukaryotes (Yamada and Sakakibara, 2018). The screening of a mouse brain library using a bait encoding the N-terminal region of Nwd1 led to the isolation of 14 putative Nwd1-binding partners, including Abcd3, Clvs2, Ets1, Paics, Quaking, and Wdr74 (Table S1). Of these binding candidates, Paics was frequently isolated as independent cDNA clones. The interaction between Nwd1 and Paics in yeast was confirmed by the co-transformation of Nwd1 with the rescued Paics plasmid (Figures 5A and 5B). Paics is a bifunctional enzyme that catalyzes *de novo* purine synthesis and is composed of two distinct enzymatic domains: 4-(*N*-succinylcarboxamide)-5-aminoimidazole ribonucleotide synthetase (SAICARs, EC 6.3.2.6) activity in its N-terminal region and 5-aminoimidazole ribonucleotide carboxylase (AIRc, EC 4.1.1.21) activity in its C-terminal region. Since all *Paics* cDNAs identified by Y2H corresponded to the C-terminal region, encompassing AIRc activity domain (Table S1), it is likely that Nwd1 binds to Paics via this domain (Figure 5C). Nwd1–Paics interaction was further verified by a co-immunoprecipitation (co-IP) experiment using HEK293 cells expressing the FLAG-tagged full-length Nwd1 (FLAG-Nwd1) and EGFP-tagged full-length Paics (Paics-EGFP). Figure 5D shows that the Paics protein was specifically co-immunoprecipitated with FLAG-Nwd1.

### Nwd1 and Paics Are Localized in Purinosomes

**Figure 6:**
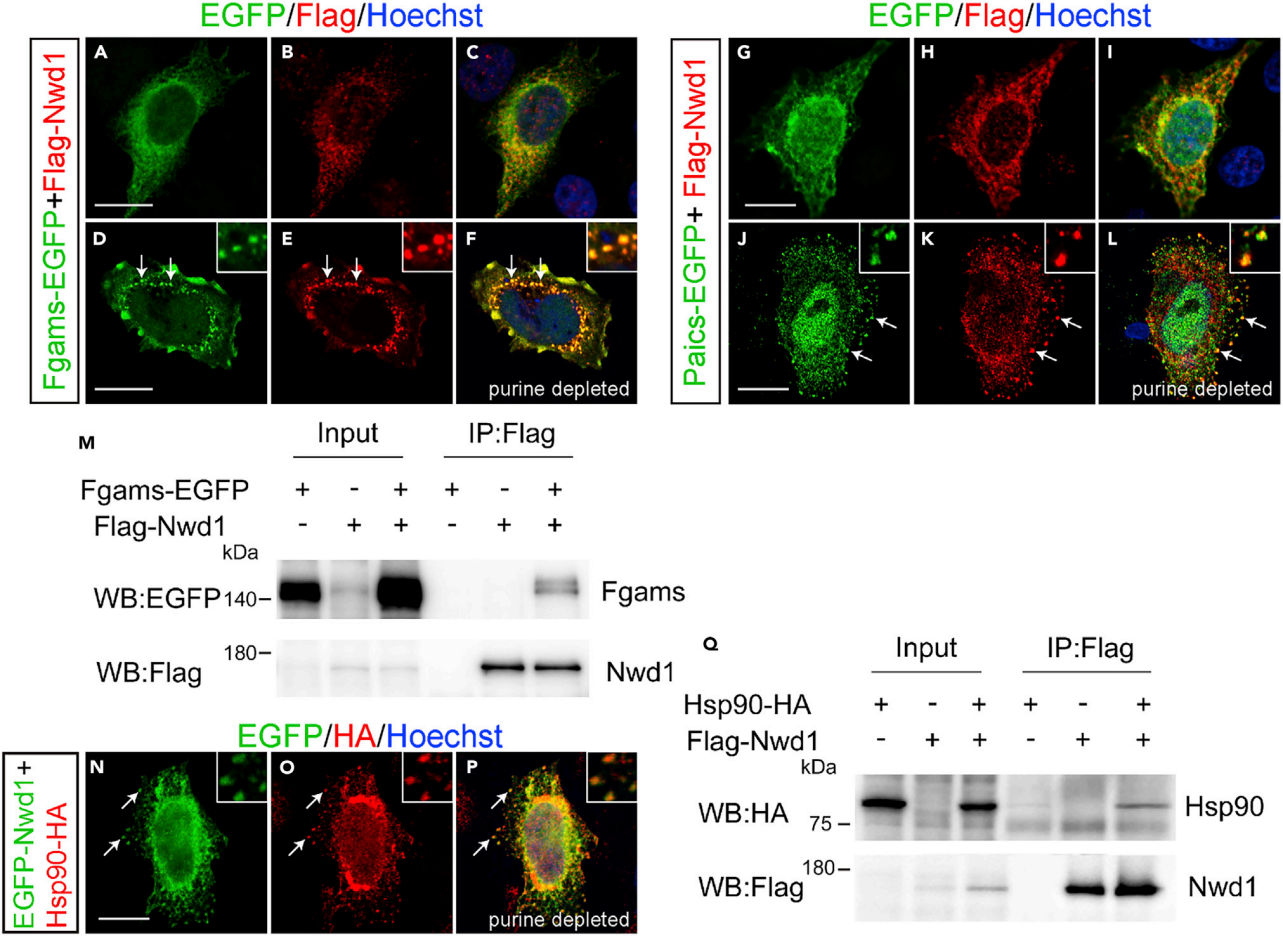
Nwd1 and Paics Are Localized in Purinosomes (A–L) HeLa cells were transfected with FLAG-Nwd1 (red) together with Fgams-EGFP (A–F) or Paics-EGFP (G–L) and cultured in complete medium (A–C and G–I) or in purine-depleted medium (D–F, J–L). Nwd1 was co-clustered with Fgams or Paics as purinosomes in purine-depleted medium (arrows). Nuclei were stained with Hoechst dye (blue). Insets in (D–F) and (J–L) showed the magnified view of the individual purinosomes formed in cytoplasm. (M) Co-IP showing the interaction between Nwd1 and Fgams. HEK293 cells expressing FLAG-Nwd1 and/or Fgams-EGFP were subjected to immunoprecipitation with an anti-FLAG antibody, followed by immunoblotting with anti-EGFP or anti-FLAG antibodies. (N–P) HeLa cells co-transfected with EGFP-Nwd1 (N) and Hsp90-HA (O) were cultured under purine-depleted conditions. (P) Merged view. The arrows indicate the colocalization of Nwd1 and Hsp90 in purinosomes. Insets showed the high power photomicrograph of the individual purinosomes. (Q) Interaction between Nwd1 and Hsp90. HEK293 cells expressing Hsp90-HA and/or FLAG-Nwd1 were subjected to immunoprecipitation using an anti-FLAG antibody, followed by immunoblotting with an anti-HA or anti-FLAG antibody. Scale bars, 15 μm.

We investigated the localization of Paics in the embryonic and postnatal mouse brain. An immunostaining analysis using an anti-Paics antibody showed high levels of Paics expression in the developing brain. At E10.5, Paics immunoreactivity was uniformly detected in the undifferentiated NSPCs in the telencephalon, extending from the VZ to the outer part of the neural tube (Figure 5E). We also noticed elevated Nwd1 expression in a small number of neurons that were β tubulin III^+^ and occupied the peripheral marginal zone (arrows in Figures 5E–5G). As CNS neurogenesis proceeds, mitotic NSPCs become restricted to the VZ and the differentiated neurons form CP. Correspondingly, at E13.5, Paics expression was observed in many β tubulin III^+^ neurons in the CP, in addition to NSPCs lining the ventricular surface (Figures 5K–5M). At E18.5, a lower but detectable Paics expression level was observed in the cerebral cortex (unpublished data). This spatiotemporal expression pattern of Paics was highly comparable with that of Nwd1 (Figures 5H–5J and 5N–5P) (Yamada and Sakakibara, 2018). Considering the equivalent distribution in the brain and its interaction with Paics, we hypothesized that Nwd1 is involved in the formation of the purinosome. To investigate the localization of Nwd1 in purinosomes, we examined the colocalization of Nwd1 with Paics or Fgams, both of which are used widely as purinosome markers. Because it is technically difficult to detect endogenously formed purinosomes, exogenously introduced markers (such as Fgams-EGFP) are generally used to label intracellular purinosomes (Pedley and Benkovic, 2017). Thus, HeLa cells that expressed FLAG-Nwd1 and Paics-EGFP or Fgams-EGFP transiently were cultured in purine-depleted media, to induce the formation of cellular purinosomes (An et al., 2008). Both the Fgams-EGFP and Paics-EGFP proteins exhibited a diffuse cytoplasmic distribution in purine-rich medium (Figures 6A–6C and 6G–6I). In the purine-depleted cells, however, many purinosomes became evident as the cytoplasmic clustering of Fgams-EGFP or Paics-EGFP (Figures 6D–6F and 6J–6L), as described previously (An et al., 2008). We observed the confined distribution and coclustering of FLAG-Nwd1 with Fgams-EGFP^+^ or Paics-EGFP^+^ purinosomes (*arrows* in Figures 6E and 6K). Such synchronously regulated coclustering of Nwd1 with Fgams and Paics during the purinosome formation allowed us to postulate that Nwd1 could also bind to Fgams. As shown in Figure 6M, we performed a co-IP experiment using HEK293 cells and demonstrated the interaction of FLAG-Nwd1 with Fgams-EGFP.

A previous study that used co-IP with FGAMS followed by a proteomics analysis demonstrated that heat shock protein 90 (Hsp90) and Hsp70 colocalize in the purinosome (French et al., 2013). Knockdown of these chaperones leads to the disruption of purinosomes, implying the involvement of Hsp90/Hsp70 chaperone machinery in the protein complex assembly (French et al., 2013). The molecular chaperones Hsp70 and Hsp90 are ubiquitously expressed proteins that have many functions, including assisting in protein folding and stabilizing protein complexes (Makhnevych and Houry, 2012); however, their exact function in purinosome formation remains unclear (Pedley and Benkovic, 2017). To examine the colocalization of Nwd1 and Hsp90 in purinosomes, the distribution of EGFP-Nwd1 and HA-tagged Hsp90 (Hsp90-HA) was assessed in purine-depleted cells; we observed overlapping localization of Nwd1 in purinosomes (arrows in Figures 6N–6P). A co-IP assay of HEK293 cells expressing FLAG-Nwd1 and Hsp90-HA demonstrated an interaction between Nwd1 and Hsp90 (Figure 6Q). Consistent with this, Correa et al. showed that NWD1 binds to HSP90 in the human prostate cancer cell line, LNCaP (Correa et al., 2014). Taken together, these results strongly indicate that Nwd1 is a component of purinosomes. Conceivably, Nwd1 may act in cooperation with the chaperone machinery in purinosome assembly or stabilization.

### Purinosome Assembly Is Regulated by Nwd1 in NSPCs

**Figure 7:**
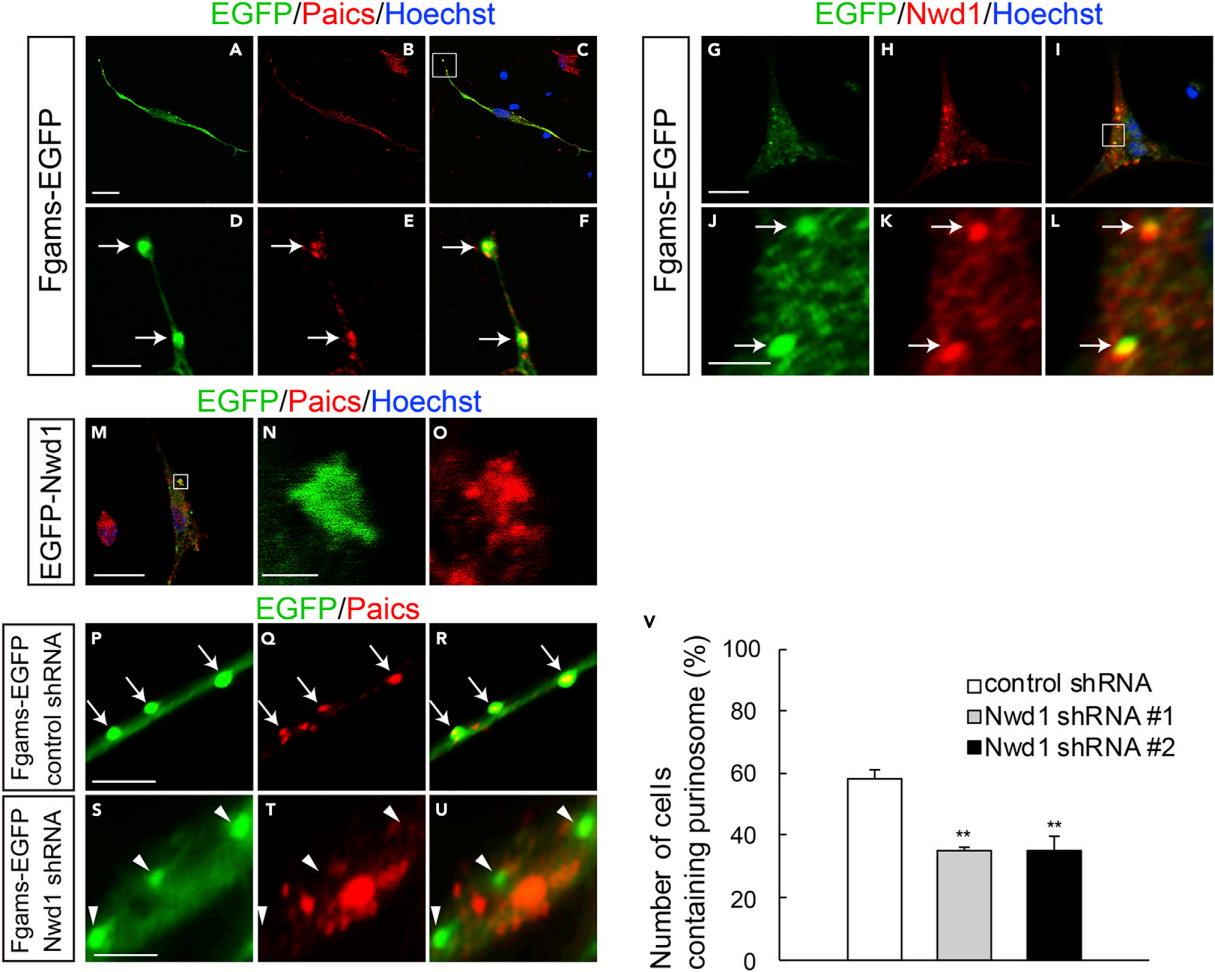
Nwd1 Regulates Purinosome Assembly in NSPCs (A–C) NSPCs derived from E12.5 telencephalons underwent electroporation with Fgams-EGFP (A) and were immunostained with an anti-Paics antibody (B, red) at 2 div. (C) Merged view. Nuclei (blue). (D–F) Higher magnification of the boxed area depicted in (C), showing the clustered signals of Fgams^+^ (D) Paics^+^ (E) purinosomes (arrows) in NSPCs. (F) Merged view. (G–L) Fgams-EGFP-expressing (G) NSPCs were immunostained with an anti-Nwd1 antibody (H). (I) Merged view. Nuclei (blue). (J–L) Higher magnification of the boxed area depicted in (I), demonstrating the localization of endogenous Nwd1 (K) in Fgams-EGFP^+^ (J) purinosomes. (L) Merged view. (M) EGFP-Nwd1-expressing NSPCs were immunostained with an anti-Paics antibody. (N and O) Higher magnification of the boxed area depicted in (M), showing the colocalization of EGFP-Nwd1 (N) and endogenous Paics (O) in purinosomes. (P–V) NSPCs were electroporated with the control shRNA (P–R) or Nwd1 shRNAs (S–U) together with Fgams-EGFP, followed by immunostaining with an anti-Paics antibody at 2 div. The arrows indicate the Fgams-EGFP^+^ Paics^+^ functional purinosomes in NSPCs. The arrowheads indicate the Fgams^+^ Paics^–^ cells. (V) Number of NSPCs containing Fgams-EGFP^+^ Paics^+^ purinosomes. Data are presented as means ± SEM. ∗∗p < 0.01, Welch's t test followed by Holm-Bonferroni correction. Scale bars, 20 μm in (A)–(C) and (G)–(I), (M); 4 μm in (D)–(F), (J)–(L), and (P)–(U); 2 μm in (N), (O).

To date, there is no evidence of the induction of purinosome assembly in nervous tissues. Therefore, next, we investigated whether NSPCs are capable of forming purinosomes and whether Nwd1 localizes in these structures in NSPCs. NSPCs isolated from the E12.5 cerebral cortex and cultured as a monolayer frequently exhibit Nestin^+^ fine unipolar or bipolar processes, resulting in a morphology that resembles that of neuroepithelial cells in the embryonic VZ (Figures S6B and S6E). The expression of Fgams-EGFP distinctly emerged as a granular structure (Figures 7A–7D). Immunostaining using the anti-Paics antibody indicated that a significant proportion of the endogenous Paics protein colocalizes in these clusters (Figures 7E, 7F, and S6G–S6R). The colocalization of Fgams and Paics, which are two enzymes that are essential for *de novo* purine biosynthesis, strongly suggested that these clusters are functional purinosomes in the NSPCs. Purinosomes were often observed within the cellular processes, in addition to the cell body, under the plasma membrane of NSPCs (Figure 7F). Immunostaining with an anti-Nwd1 antibody revealed the localization of the endogenous Nwd1 protein in Fgams-EGFP^+^ purinosomes in NSPCs (Figures 7G–7L). The purinosome localization of Nwd1 became more evident after the introduction of EGFP-Nwd1 into NSPCs (Figure 7N). These data showed the presence of purinosomes in NSPCs.

To examine the role of Nwd1 in purinosome assembly, Nwd1 expression was suppressed by shRNA in NSPCs. *Nwd1* shRNA constructs were electroporated into NSPCs expressing Fgams-EGFP. At 2 div, we counted the number of cells containing the functional purinosomes that are defined as the granules simultaneously labeled with Fgams-EGFP and endogenous Paics. As shown in Figure 7V, compared with the non-targeting shRNA (Figures 7P–7R), *Nwd1* shRNAs reduced the number of cells containing Fgams-EGFP^+^ Pacis^+^ purinosomes considerably (Figures 7S–7U) (control, 58.3 ± 3.0%; shRNA #1, 35.0 ± 1.5%; shRNA #2, 34.7 ± 4.8%). We noticed that *Nwd1* KD resulted in a decreased number of Paics^+^ granules in NSPCs, whereas the total number of exogenously introduced Fgams-EGFP^+^ granules remained unchanged (Figure S6S). Consistent with this, the fraction of cells that were labeled with Fgams-EGFP alone (Fgams-EGFP^+^ Paics^–^) was increased in NSPCs upon treatment with the shRNAs (arrowheads in Figure 7U). Because a protein complex lacking Paics no longer functions as a purinosome, we concluded that Nwd1 is required for the assembly of the functional purinosome in NSPCs.

### Purinosome Enzymes Are Essential for Cortical Development

**Figure 8:**
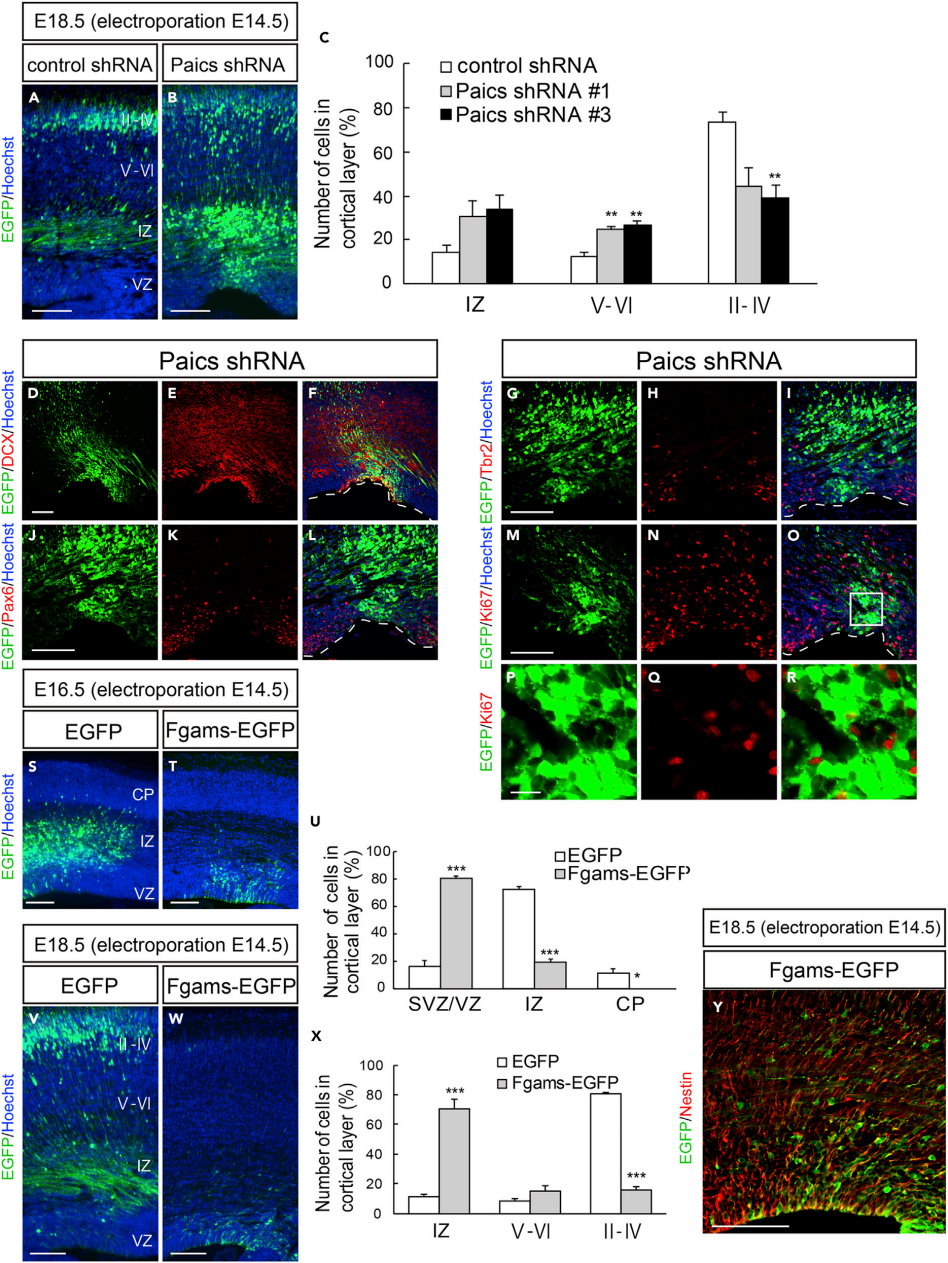
Purinosome Components Regulate Cortical Development (A–R) A control shRNA or Paics shRNA (shRNA #1 or shRNA #3) was delivered into the brain on E14.5, together with EGFP, and the cortices were analyzed at E18.5. (A and B) Distribution of EGFP^+^ cells in the neocortex. (C) Quantification of the distribution of EGFP^+^ cells in the indicated areas. ∗∗p < 0.01. (D–R) Brain sections of *Paics* knockdown were immunostained using anti-Dcx (D–F), anti-Pax6 (J–L), anti-Tbr2 (G–I), and anti-Ki67 (M–O) antibodies. The VZ surface is outlined by the dashed line. (P–R) Higher magnification of the boxed area depicted in (O). Many NSPCs electroporated with the *Paics* shRNAs remained in the SVZ/VZ/IZ as postmitotic cells that were Dcx^+^ Pax6^–^ Tbr2^–^. (S–Y) Control EGFP or Fgams-EGFP was delivered into the brain on E14.5, and the cortices were analyzed at E16.5 (S and T) or E18.5 (V, W, and Y). (U and X) Distribution of EGFP^+^ cells in the indicated areas at E16.5 (U) or E18.5 (X). ∗p < 0.05, ∗∗∗p < 0.001. (Y) Fgams-EGFP-overexpressing brain immunostained with an anti-Nestin antibody. Forced expression of Fgams-EGFP directed many NSPCs to remain in the SVZ/VZ as Nestin^+^ cells, and only a few cells reached the border between the SVZ and IZ. All data are presented as means ± SEM. Statistical significance value was determined using the Welch's t test followed by Holm-Bonferroni correction. Scale bars, 100 μm in (A)–(O) and (S)–(Y); 15 μm in (P)–(R).

To clarify the involvement of the purinosome in brain development, we examined the loss-of-function or gain-of-function phenotypes of Paics and Fgams. First, E14.5 embryos were electroporated *in utero* with *Paics* shRNAs to knockdown the expression of endogenous Paics (Figure S7A). As shown in Figure 8B, *Paics* shRNAs significantly repressed neuronal migration in the neocortex at least until 4 days post electroporation (E18.5). Compared with the non-targeting control shRNA, *Paics* KD resulted in a decreased number of neurons that reached the upper layers II–IV (control shRNA, 73.3 ± 4.8%, n = 8; *Paics* shRNA #1, 44.2 ± 8.2%, n = 4; *Paics* shRNA #3, 39.3 ± 5.8%, n = 9) (Figures 8A–8C). Instead, a considerable number of cells accumulated within the IZ and lower cortical layers (V–VI) of embryos expressing *Paics* shRNAs (IZ: control shRNA, 14.4 ± 3.3%, n = 8; *Paics* shRNA #1, 30.7 ± 7.3%, n = 4; *Paics* shRNA #3, 34.0 ± 6.1%, n = 9; layers V–VI: control shRNA, 12.3 ± 2.4%; *Paics* shRNA #1, 25.1 ± 1.2%; *Paics* shRNA #3, 26.8 ± 2.0%; Figure 8C). Notably, 4 days after *Paics* KD, a significant number of *Paics* KD cells persisted in the VZ/SVZ (Figure 8D). Immunostaining analysis revealed that these VZ/SVZ cells were Dcx^+^, Tbr2^–^, and Pax6^–^ (Dcx^+^: control shRNA, 13.6 ± 3.7%, n = 7; *Paics* shRNA, 70.5 ± 4.4%, n = 4; Tbr2^+^: control shRNA, 17.9 ± 2.4%, n = 4; *Paics* shRNA, 4.5 ± 2.1%, n = 4; Pax6^+^: control shRNA, 47.2 ± 3.0%, n = 5; and *Paics* shRNA, 20.5 ± 10.0%, n = 4) (Figures 8D–8L and S8A). Consistently, most of these cells were negative for Ki67 (control shRNA, 29.6 ± 5.8%, n = 5; *Paics* shRNA, 7.5 ± 2.0%, n = 4) (Figures 8M–8R and S8A), indicating that Paics loss of function induced mitotic exit and premature differentiation of NSPCs.

**Figure 9:**
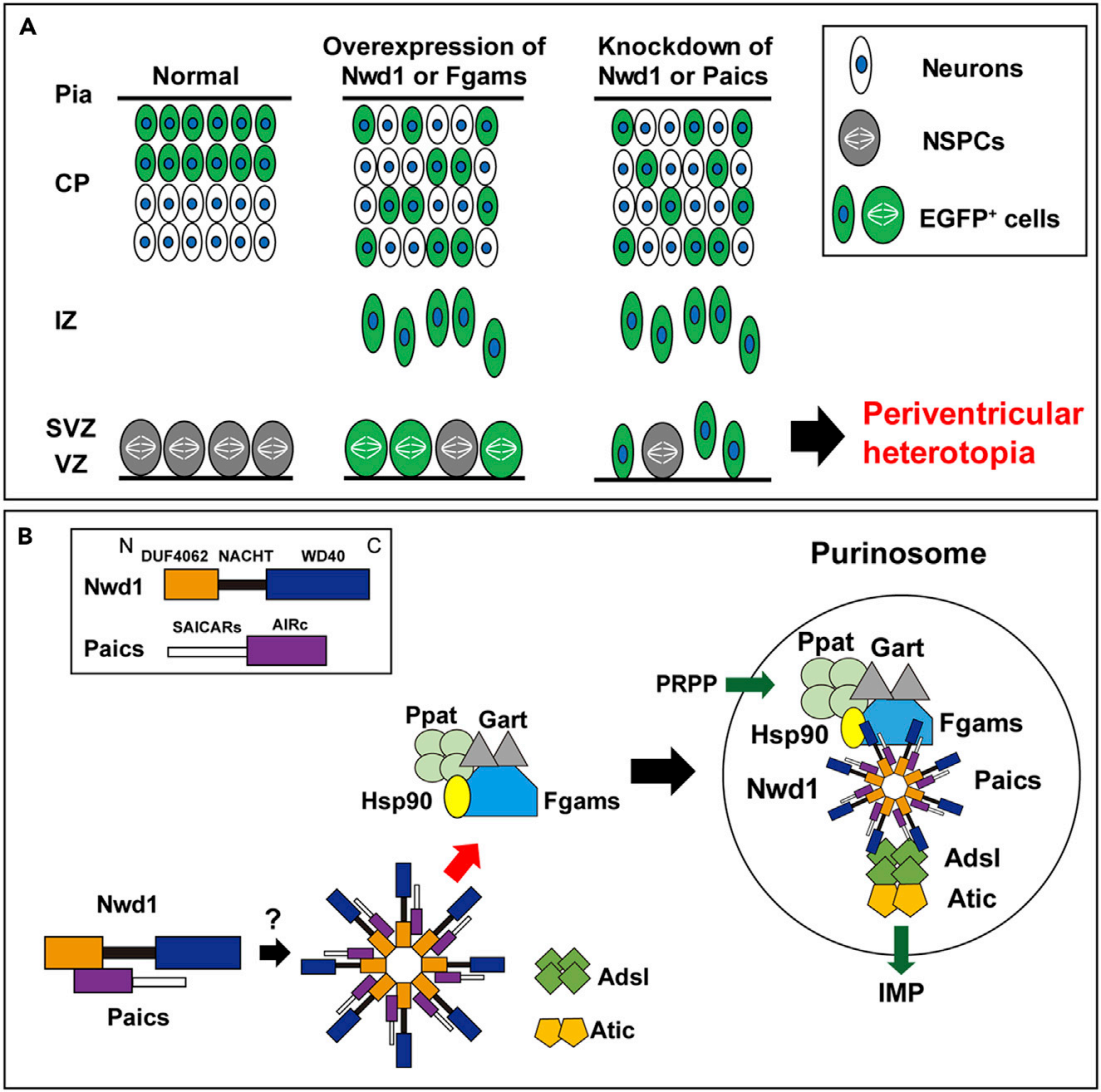
Models of Nwd1 Function in Corticogenesis and in Purinosome Formation (A) Purinosome assembly/disassembly regulates cortical development. Overexpression of purinosome components (Nwd1 or Fgams) disturbs the radial migration of neurons and causes a significant increase in the Nestin^+^ NSPC pool in the VZ/SVZ. Knockdown of purinosome components (Nwd1 or Paics) accelerates mitotic exit and premature differentiation of NSPCs and represses neuronal migration leading to periventricular heterotopia. EGFP^+^ cells represent cells harboring the transgene. (B) Hypothetical molecular model of purinosome formation by Nwd1. Undiscovered signals trigger the interaction of Nwd1 with Paics via the DUF4062 and AIRc domains, mediating the formation of the multimeric structure of Nwd1–Paics. The Nwd1–Paics complex systematically tethers other core enzymes, including Fgams, Ppat, and Gart, to form a functional purinosome. Nwd1, NACHT and WD repeat domain-containing protein 1; Ppat, phosphoribosyl pyrophosphate amidotransferase; Gart, phosphoribosylglycinamide formyltransferase; Fgams, formylglycin-amidine ribonucleotide synthase; Paics, phosphoribosylaminoimidazole carboxylase phosphoribosylaminoimidazole succinocarboxamide synthetase; Adsl, adenylosuccinate lyase; Atic, 5-aminoimidazole-4-carboxamide ribonucleotide formyltransferase inosine monophosphate cyclohydrolase; PRPP, phosphoribosyl diphosphate; IMP, inosine monophosphate; Hsp90, heat shock protein 90.

Next, we assessed the effect of Fgams overexpression on neurogenesis and neuronal migration *in vivo*. *Fgams*-EGFP was introduced into NSPCs *in utero* at E14.5. Fgams overexpression significantly suppressed neuronal migration from the VZ, leading to the accumulation of Fgams-overexpressing cells in the VZ/SVZ at E16.5 (control, 16.5 ± 4.2%, n = 6; Fgams, 80.3 ± 1.9%, n = 7) (Figures 8S–8U). Fgams-overexpressing cells were rarely observed within the IZ (control, 72.3 ± 2.5%; Fgams, 19.7 ± 1.9%). At E18.5, most Fgams-EGFP^+^ cells remained in the IZ and SVZ (IZ: control, 11.3 ± 1.8%, n = 4; Fgams, 70.9 ± 5.9%, n = 8) and fewer cells were found in the cortical neuron layers (Figures 8W and 8X). The Fgams-EGFP^+^ cells that accumulated in the germinal area were Nestin^+^ (EGFP, 29.0 ± 6.0%, n = 12; Fgams-EGFP, 57.2 ± 6.5%, n = 4) (Figures 8Y and S8B–S8E), suggesting that they were undifferentiated NSPCs. Taken together, these data provide strong evidence that both Paics and Fgams are essential for neurogenesis and corticogenesis and that the dysregulation of the genes that encode these proteins hinders neuronal migration. Such abnormal properties of neurons and NSPCs caused by the manipulation of Paics and Fgams seemed to be a phenocopy of Nwd1 overexpression/knockdown (Figures 1 and 2). It is likely that the *de novo* biosynthesis of purines, especially the tightly regulated levels of purinosome components, is indispensable for the orchestrated migration and differentiation of neurons that occur during brain development (Figure 9A).

## Discussion

### Nwd1 as a Novel Component of Purinosomes

Here, we described the induction of the formation of Fgams^+^ Paics^+^ purinosomes in NSPCs. We also revealed that Nwd1 interacts with Paics and is localized in purinosomes in NSPCs. Nwd1 functions as a component in the assembly of purinosomes. Nevertheless, Nwd1 has no enzymatic activity related to purine biosynthesis, unlike Paics and Fgams. It is possible that Nwd1 participates in the assembly of purinosomes as a member of the STAND family of proteins. The STAND proteins are a newly recognized ATPases associated with diverse cellular activities (AAA) type of ATPases that act as signaling hubs and mediate the energy-dependent remodeling of proteins and the translocation of macromolecules. Generally, *STAND* genes encode multidomain proteins, typically encompassing an N-terminal effector domain, a centrally located NACHT domain that has P loop ATPase activity and mediates self-oligomerization, and a C-terminal ligand-binding domain (Leipe et al., 2004). The binding of specific ligands onto the C-terminal domain elicits a conformational change in STAND proteins, which is dependent on ATP levels; this results in the formation of the oligomeric ring-shaped superstructures of STAND proteins, which exhibit a central pore (Mermigka et al., 2020). Such superstructures serve as the tightly regulated molecular switch that controls diverse biological processes, including apoptosis and innate immune responses, in which the ring-like superstructures of STAND proteins drive the translocation or remodeling of the substrate proteins (Mermigka et al., 2020).

Among the STAND-family proteins, Nwd1 shares a similar domain structure with Apaf1 (Dorstyn et al., 2018, Leipe et al., 2004, Yamada and Sakakibara, 2018). During apoptosis, the C-terminal WD40 domain of Apaf1 binds to the cytochrome *c* molecules that leaked from damaged mitochondria. This ligand binding induces the energy-dependent self-oligomerization of Apaf1. Subsequently, the ring-like superstructure of Apaf1 tethers caspase 9 through the N-terminal CARD domain of Apaf1 to form the macromolecular complex named apoptosome, which triggers the apoptotic caspase cascade (Zou et al., 1999). Similarly, Paics is assembled as a homo-octameric structure in purinosomes (Li et al., 2007), similar to the caspase 9 heptamer in apoptosomes. Based on these observations, we postulated that Nwd1 undergoes an ATP-dependent conformational change upon binding to the ligand(s), via which Paics proteins are triggered to be recruited to a purinosome (Figure 9B). Primary complex of Nwd1/Paics and several chaperones including Hsp90 might systematically tether other core enzymes, including Fgams, Ppat, and Gart to form an integrated purinosome (Figure 9B). This complicated molecular machinery may explain why the *in vitro* reconstitution of a functional purinosome has been unsuccessful thus far. Nwd1 may act as a sensor protein that drives the assembly and disassembly of purinosomes and activates the *de novo* purine biosynthesis pathway during CNS development.

### Purinosome Components Regulate the Maintenance of NSPCs and Neuronal Migration during Cortical Development

We reported previously the strong expression of Nwd1 in NSPCs and immature neurons during the development of the rodent brain (Yamada and Sakakibara, 2018). The present study revealed a similar distribution of Paics and Nwd1 in the developing neocortex. The gain and loss of function of Nwd1, Paics, and Fgams, which were achieved using *in utero* electroporation, demonstrated that these purinosome components are essential for proper cortical development and that their dysregulation leads to a severe delay in the migration of immature neurons (Figure 9A). In addition, *in vivo* knockdown of Nwd1 resulted in a decrease in the number of Pax6^+^ apical progenitors, in conjunction with the ectopic emergence of Tbr2^+^ basal progenitor cells in the embryonic VZ. A previous study reported that the forced expression of the Tbr2 transcription factor directs the conversion of radial glia into basal progenitor cells (Sessa et al., 2008). Thus, we assumed that the altered expression level of Nwd1 caused the premature differentiation of NSPCs, suggesting a vital role for this protein in the maintenance of NSPC pools, including CNS stem cells (Figure 9A). Consistently, a previous study suggested a possible role for Nwd1 in tumor cells endowed with stem-cell-like properties; i.e., the proliferative and self-renewing properties. The expression of Nwd1is strikingly upregulated by Sox9, a transcription factor in malignant prostate tumor cells (Correa et al., 2014). A gain- and loss-of-function study indicated that Sox9 plays a central role in the specification and maintenance of CNS stem cells that reside in the embryonic VZ and adult SVZ (Scott et al., 2010). As a downstream target of Sox9, Nwd1 may have a function in the maintenance of CNS stem cells. Interestingly, it was also demonstrated that Paics is necessary for the proliferation and invasion of prostate cancer cells and that the silencing of Paics expression abrogates the progression of several types of prostate tumors (Chakravarthi et al., 2018). Taken together with this evidence, our findings imply that the formation of the purinosome machinery is crucial for the maintenance of somatic stem cells and tumor cells, which commonly require a large amount of *de novo* purine production.

In addition, we demonstrated that a tight control of the level of expression of Nwd1 is crucial for neurite extension and axon formation and that altered levels of expression of the *Nwd1* gene caused migration defects in cortical neurons *in vivo*. Considering that the spatiotemporally controlled outgrowth of neurites is needed for the establishment of neuronal polarity and neuronal migration (Hansen et al., 2017), purinosome formation might be closely linked to the dynamic morphological transformation of migrating neurons that occurs during corticogenesis. Purines affect many aspects of neuronal differentiation. For example, the activation of Rac, which is a small GTP-binding protein, is required for the formation of the leading process in radially migrating neurons in the embryonic cerebral cortex (Konno et al., 2005). Nwd1 might affect discrete aspects of neural development, including neuronal migration and the maintenance of the NSPC pool, via the regulation of the assembly/disassembly of purinosomes.

However, *de novo* purine synthesis is energy intensive and required for numerous substrates; therefore, it has been thought that terminally differentiated neurons place greater reliance on the purine salvage pathway than the *de novo* pathway to achieve prompt repair of damaged DNA and prevent neurodegeneration. A previous *in vitro* quantitative analysis of purines using neuroblastoma cell lines demonstrated that the intracellular purine content increases as neuronal differentiation proceeds, whereas *de novo* purine synthesis decreases during neuronal differentiation (Göttle et al., 2013), suggesting that a regulated balance between the *de novo* and purine salvage pathway is critical for coordinated neuronal differentiation. However, the switching molecules that determine this balance remain unidentified. Our current study provides an insight into the machinery governing purine metabolism during nervous system development.

### Implication of Nwd1 and Purinosome Components in Neurological Disorders

Downregulation of Nwd1 by shRNA expression in the embryonic cerebral cortex often caused the cortical dysgenesis similar to human periventricular nodular heterotopia (PH), a cortical malformation that is characterized by the formation of ectopic aggregates of neurons that line the lateral ventricle. These nodules exhibited a rosette-like structure and were filled with the glutamatergic neurons innervated by VgulT1^+^ excitatory terminals, indicating the formation of abnormal excitatory circuit. In humans, PH is associated with intractable epilepsy and intellectual disability (Cossu et al., 2018). Previous studies using a genetic animal model showed that PH is caused by the failure of the radial migration of newborn neurons from the VZ in addition to the abnormal proliferation of NSPCs (Li et al., 2015, Lian and Sheen, 2015); however, the molecular mechanisms underlying the development of PH are not fully understood. Thus, the disturbance of the purine *de novo* synthesis pathway may be associated, at least in part, with the mechanism underlying the pathogenesis of PH.

In addition to PH, purinosome-related genes are responsible for certain neurological disorders. Deficiency of ADSL in humans causes atrophy of distinct regions of the brain, including the cerebral cortex, in addition to hypomyelination and lissencephaly (Jurecka et al., 2015). Patients with ATIC mutation exhibit neurological symptoms, including profound mental retardation and epilepsy accompanied by various dysmorphic features (Marie et al., 2004). A previous study that used cultured fibroblasts from these patients demonstrated that ATIC and ADSL mutations destabilize the assembly of the purinosome to various degrees and that the ability to form purinosomes is correlated with the severity of the phenotype of individual patients (Baresova et al., 2012). Recently, PAICS deficiency was reported in humans. Patients carrying a homozygous missense mutation in the *PAICS* gene exhibit multiple severe malformations, including a small body and craniofacial dysmorphism, resulting in early neonatal death (Pelet et al., 2019). Although inactivating mutations in the human *NWD1* gene have not been reported to date, it was recently shown that the neuronal expression of NWD1 is upregulated in patients with temporal lobe epilepsy (Yang et al., 2019). Using a mouse model of acute epileptic seizures, it was suggested that Nwd1 regulates the neuronal hyperexcitability of glutamatergic synaptic transmission in the adult brain (Yang et al., 2019). Therefore, Nwd1 might be involved in a mechanism of regulation of the synaptic transmission via the formation of purinosomes or other macromolecular complexes.

### Limitations of the Study

In the present study, we revealed that Nwd1 interacts with Paics to regulate purinosome assembly in NSPCs, whereas the formation of purinosomes in terminally differentiated neurons has not been demonstrated *in vitro* and *in vivo*. We are currently analyzing the function of purinosomes in differentiated neurons. These findings will be published in our future study. In addition, there is no clear evidence for the mechanism underlying the premature differentiation of NSPCs induced by Nwd1 gene silencing. Because the *de novo* purine synthesis pathway is generally upregulated under cellular conditions that demand higher levels of purines, such as tumor growth, it might be reasonable to suppose that the exhaustion of the purine pool in dividing NSPCs leads them to exit the cell cycle. Such an unexpected quiescent status among NSPCs may provoke the induction of fate-determining genes, such as Ascl1 and Nkx2-1, resulting in premature neuronal differentiation.

## Methods

All methods can be found in the accompanying Transparent Methods supplemental file.

## Supplemental Information

Document S1. Transparent Methods, Figures S1–S8, and Table S1
