# Supplementary material for: Nwd1 Regulates Neuronal Differentiation and Migration through Purinosome Formation in the Developing Cerebral Cortex
Source: iScience. 2020 Apr 13;23(5):101058. doi: 10.1016/j.isci.2020.101058 (PMC7186558; doi:10.1016/j.isci.2020.101058)
Supplement: Document S1. Transparent Methods, Figures S1–S8, and Table S1 [file mmc1.pdf]

iScience, Volume *23*

Supplemental Information

Nwd1 Regulates Neuronal Differentiation and Migration through Purinosome Formation in the Developing Cerebral Cortex

Seiya Yamada, Ayaka Sato, and Shin-ichi Sakakibara

Figure S1

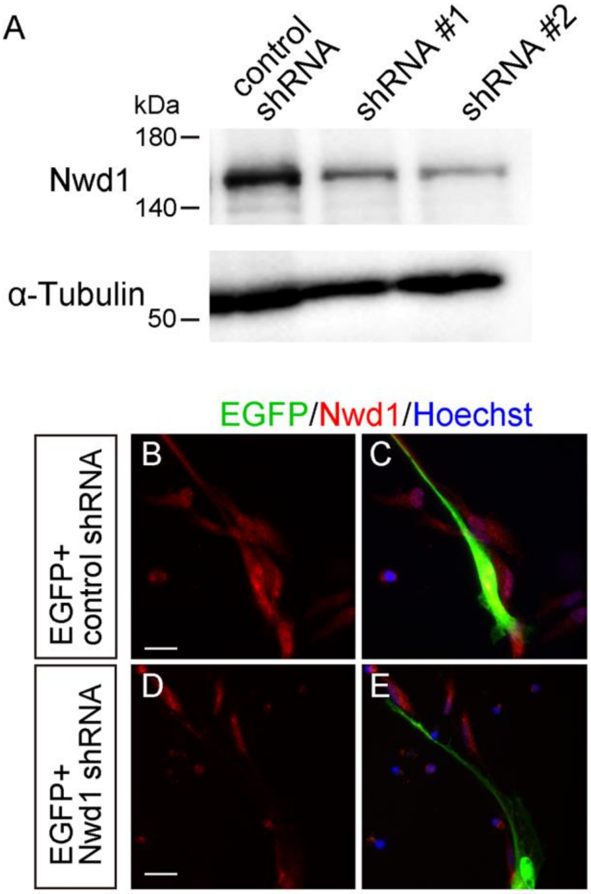

Figure S1. Validation of the *Nwd1* shRNAs. Related to Figure 1.

(A) The non-targeting control or *Nwd1* shRNA (shRNA #1 or shRNA #2) was transfected into HEK293 cells that expressed EGFP-Nwd1 exogenously. The expression level of Nwd1 was evaluated by immunoblotting using an anti-Nwd1 antibody. Immunoblotting using an α-tubulin antibody was performed to ensure the equal loading of cell lysates. (B–E) The control shRNA or *Nwd1* shRNA #1 was transfected into the primary culture of NSPCs, together with EGFP. Cells were immunostained with an anti-Nwd1 antibody at 2 div, which showed that the expression of endogenous Nwd1 (red) was silenced by the *Nwd1* shRNA. Scale bars, 20 μm.

Figure S2

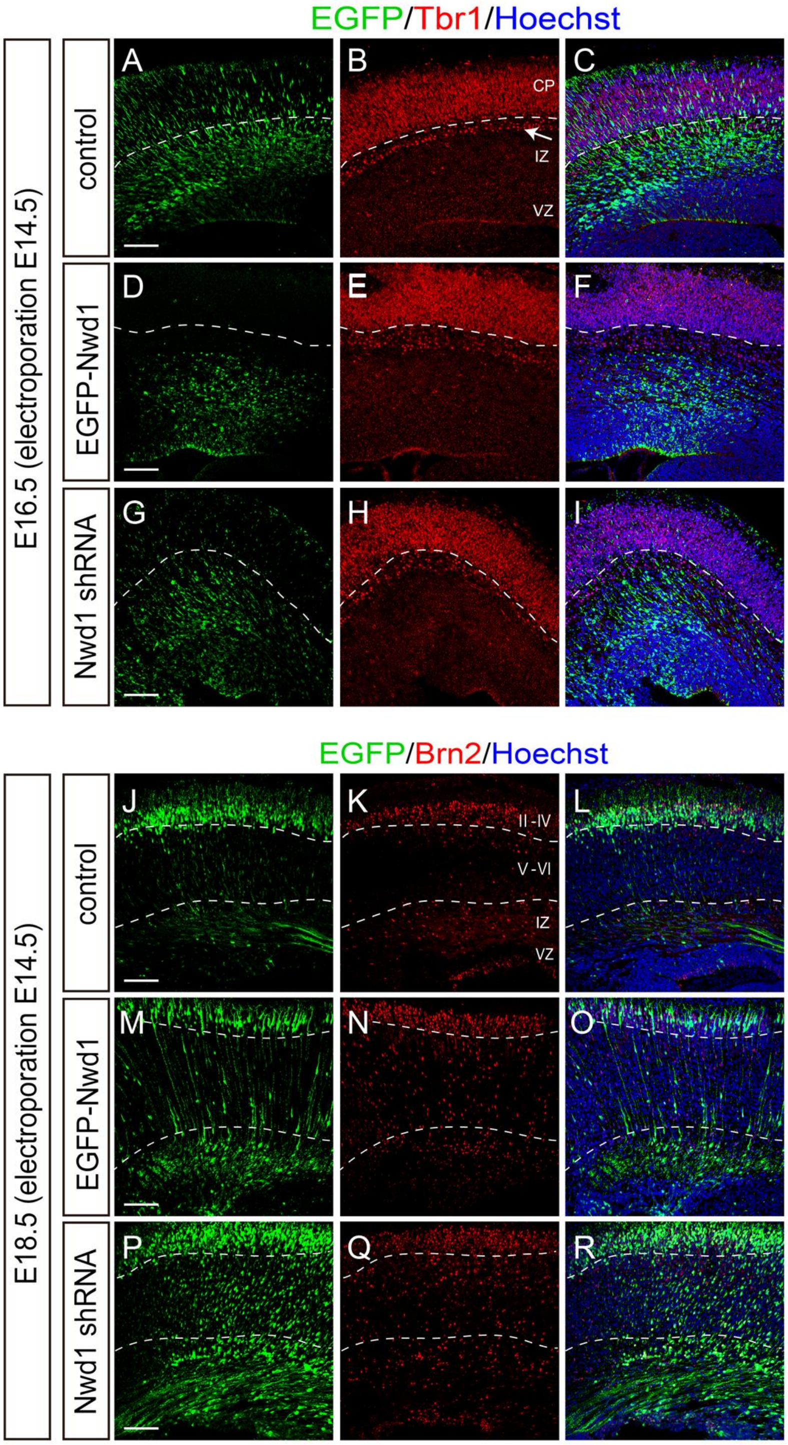
EGFP/Tbr1/Hoechst
E16.5 (electroporation E14.5)
control
EGFP-Nwd1
Nwd1 shRNA
A
B
C
D
E
F
G
H
I
CP
IZ
VZ
EGFP/Brn2/Hoechst
E18.5 (electroporation E14.5)
control
EGFP-Nwd1
Nwd1 shRNA
J
K
L
M
N
O
P
Q
R
II-IV
V-VI
IZ
VZ

Figure S2. Dysregulated expression of Nwd1 suppresses the neuronal migration. Related to Figure 1.

E14.5 embryos were electroporated *in utero* with non-targeting control (A–C, J–L), EGFP-Nwd1 (D–F, M–O) or *Nwd1* shRNA #1 (G–I, P–R) or, and brains were harvested at E16.5 (A–I) and E18.5 (J–R). The shRNA constructs were co-electroporated with EGFP. (A–I) E16.5 brain sections were immunostained with anti-Tbr1 antibody (red), a marker for the deep cortical layer and subplate. Dashed lines represent the border between CP and subplate. Arrow in (B) indicates the $Tbr1^{+}$ subplate. (J–R) E18.5 brain sections were immunostained with anti-Brn2 antibody (red), a marker for the upper cortical layers. Dashed lines denote the borders of upper layers (II–IV), deep cortical layers (V–VI) and IZ. Scale bars, 100 μm.

Figure S3

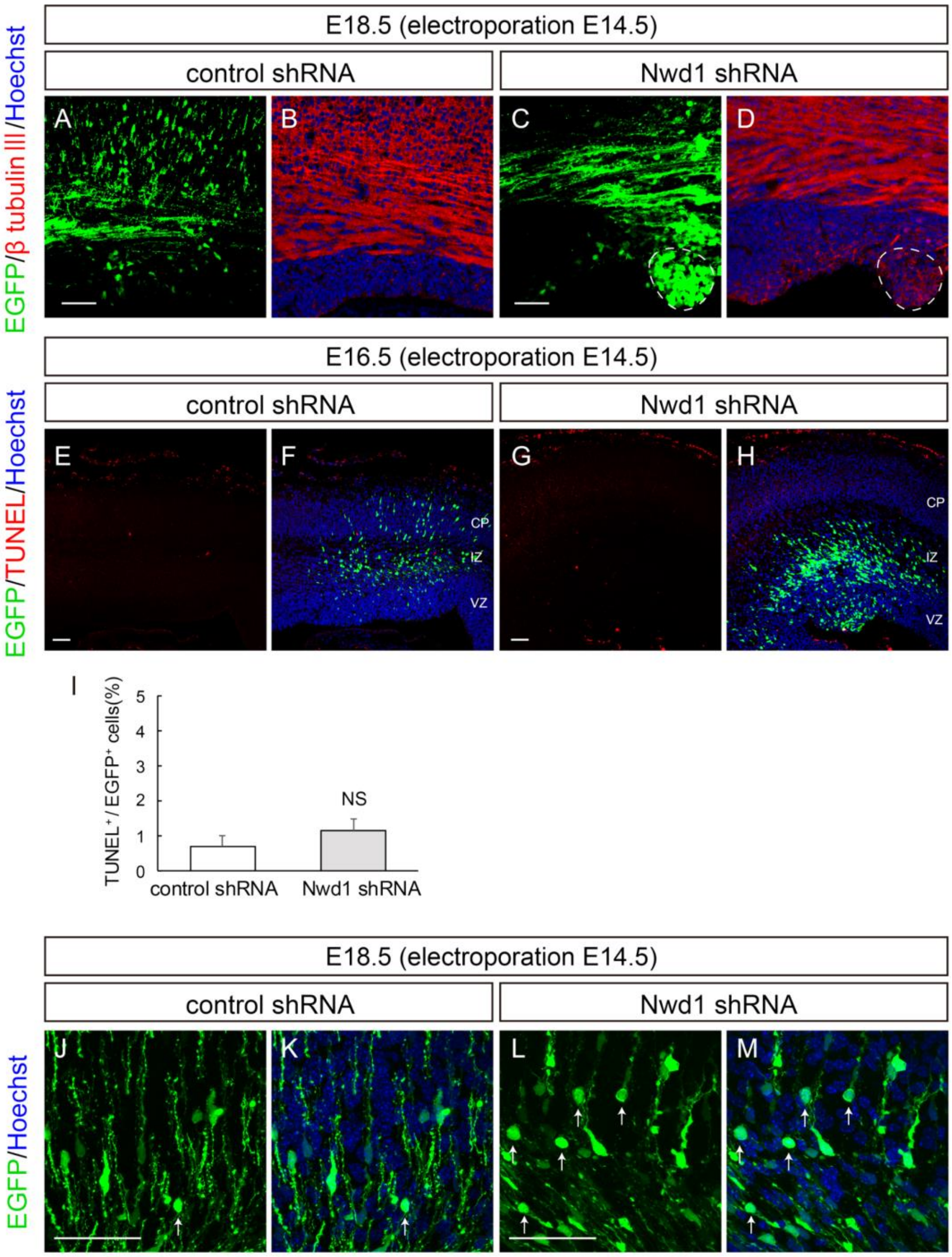

E18.5 (electroporation E14.5)
control shRNA
Nwd1 shRNA
EGFP/β tubulin III/Hoechst
A
B
C
D
E16.5 (electroporation E14.5)
control shRNA
Nwd1 shRNA
EGFP/TUNEL/Hoechst
E
F
G
H
CP
IZ
VZ
I
TUNEL+ / EGFP+ cells(%)
0
1
2
3
4
5
NS
control shRNA
Nwd1 shRNA
E18.5 (electroporation E14.5)
control shRNA
Nwd1 shRNA
EGFP/Hoechst
J
K
L
M

Figure S3. Nwd1 knockdown causes premature differentiation of NSPCs. Related to Figure 2.

The non-targeting control or *Nwd1* shRNA #1 were electroporated into E14.5 brains along with EGFP, and embryos were harvested at E16.5 and E18.5. (A–D) Confocal images of a neocortex at E18.5 stained with an anti-β-tubulin III (red) antibody. The areas surrounded by dashed line denote the distribution of cells electroporated with the *Nwd1* shRNA (green) within the VZ. (E-I) Apoptotic cells were detected by TUNEL staining (red) at E16.5. (I) Number of $EGFP^+$ $TUNEL^+$ apoptotic cells. Control shRNA, n=8; Nwd1 shRNA, n=8. Data are presented as means ± SEM. NS, not significant, Welch's *t*-test. (J–M) Confocal projection images of E18.5 cerebral cortex. Note that the significant number of Nwd1 KD cells exhibited an apolar and round morphology (arrows) within the IZ. Nuclei are counterstained with Hoechst dye (blue). Scale bars, 50 μm.

Figure S4

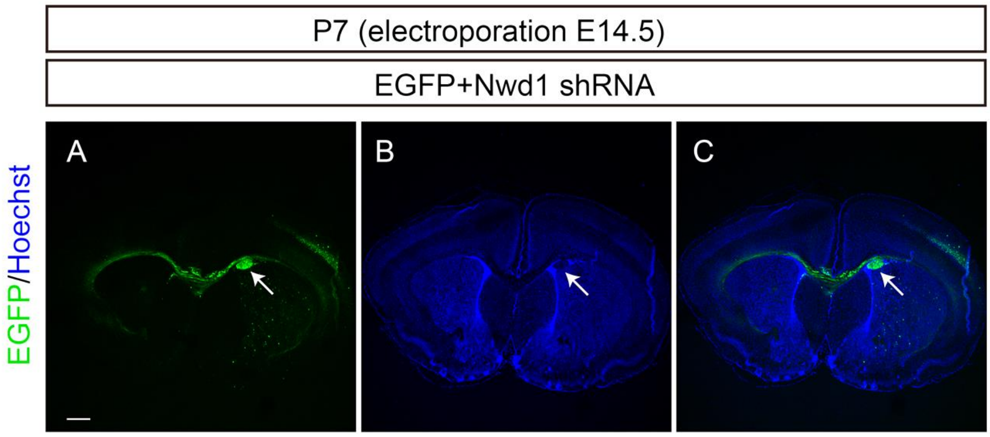

Figure S4. Periventricular heterotopia caused by Nwd1 knockdown. Related to Figure 3.

The *Nwd1* shRNA was electroporated into the neocortex at E14.5 together with EGFP, and brains were collected at P7. Representative confocal image of a coronal section, showing the ectopic formation of periventricular heterotopia composed of densely packed $EGFP^{+}$ cells (A) located under the neocortex (arrow) in the electroporated hemisphere. Nuclei were stained with Hoechst dye (B). Scale bar, 500 µm.

Figure S5

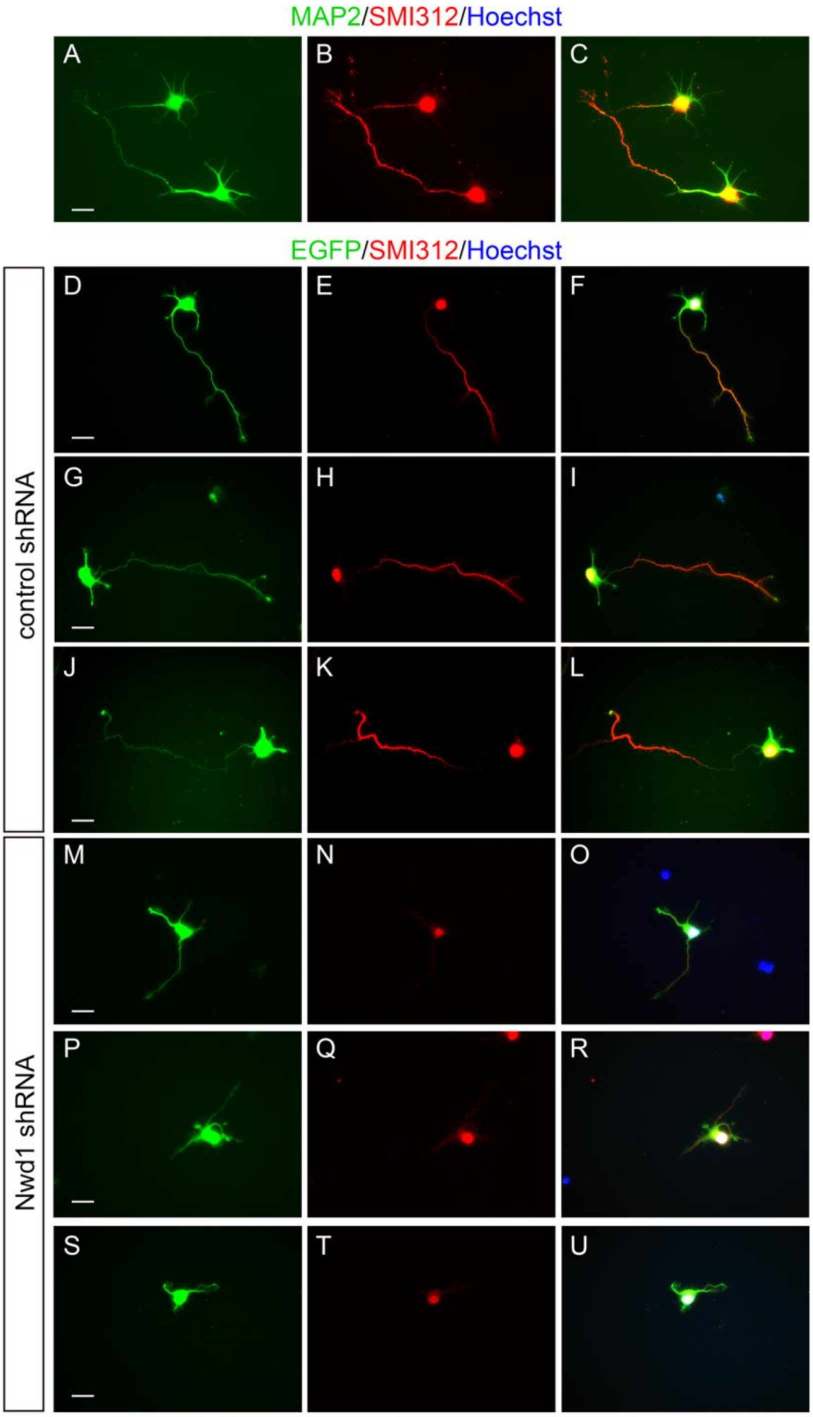

MAP2/SMI312/Hoechst
A
B
C
EGFP/SMI312/Hoechst
control shRNA
D
E
F
G
H
I
J
K
L
Nwd1 shRNA
M
N
O
P
Q
R
S
T
U

Figure S5. Nwd1 expression is required for axonal extension, Related to Figure 4

(A–C) Primary cortical neurons prepared from E16.5 embryos were cultured for 3 days (3 div) and double-immunostained with a dendritic marker, MAP2 (green), and an axon marker, SMI312 (red), confirming dendritic arborization and the extension of a single axon from differentiated neurons. (D–U) Non-targeting shRNA or *Nwd1* shRNA were electroporated together with EGFP into the cortical neurons, and each cell was immunostained with an SMI312 antibody (red) at 3 div. Inhibition of Nwd1 expression impaired axonal extension from immature neurons. Scale bars, 20 μm.

Figure S6

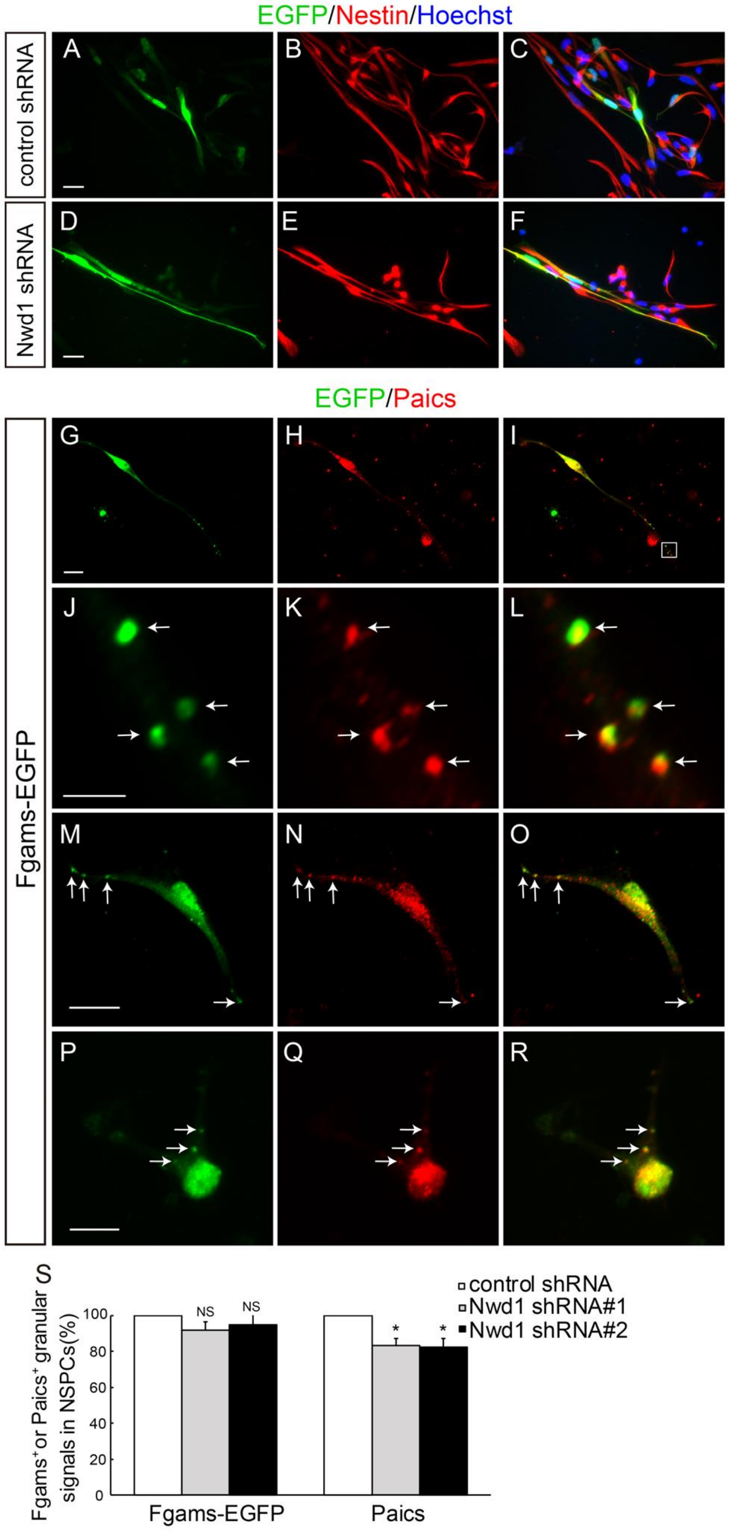

EGFP/Nestin/Hoechst
control shRNA
Nwd1 shRNA
A
B
C
D
E
F
EGFP/Paics
Fgams-EGFP
G
H
I
J
K
L
M
N
O
P
Q
R
S
Fgams⁺or Paics⁺granular signals in NSPCs(%)
100
80
60
40
20
0
NS
NS
*
*
Fgams-EGFP
Paics
control shRNA
Nwd1 shRNA#1
Nwd1 shRNA#2

Figure S6. Purinosome formation in NSPCs. Related to Figure 7.

(A–F) Primary cultured NSPCs prepared from E12.5 telencephalons were electroporated with non-targeting shRNA or *Nwd1* shRNA, together with EGFP and then immunostained with Nestin antibody (red). (G–R) NSPCs electroporated with Fgams-EGFP were immunostained with anti-Paics antibody (red). Each panel shows the high-power view of individual Fgams-EGFP$^+$ cells. (J–L) The higher magnification of the boxed area in (I) represents the formation of functional purinosomes that emerged as four clusters positive for Fgams-EGFP$^+$ and endogenous Paics$^+$ (arrows) in the distal region of the NSPC process. (M–R) Arrows indicate Fgams-EGFP$^+$ Paics$^+$ purinosomes emerged in cell process of NSPCs. (S) NSPCs were electroporated with Fgams-EGFP together with control or Nwd1 shRNA, followed by immunostaining with anti-Paics antibody at 2 div. The graph shows the number of Fgams-EGFP$^+$ or Paics$^+$ granular signals in NSPCs. Data are presented as means ± SEM. NS, not significant, $*p<0.05$, Welch's *t*-test followed by Holm–Bonferroni correction. Scale bars, 20 μm in A–F and G–I, M–R; 4 μm in J–L.

Figure S7

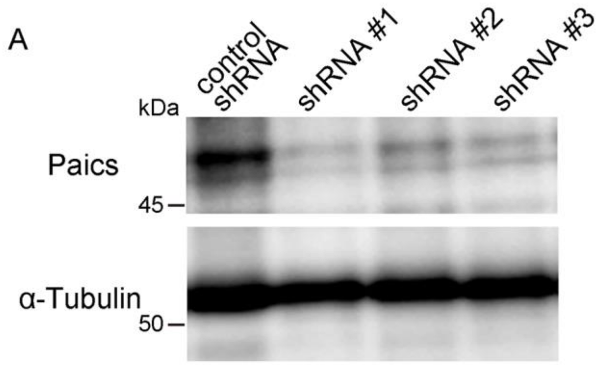

Figure S7. Validation of *Paics* shRNAs. Related to Figure 8.

N2a cells were transfected with three different mouse *Paics* shRNA constructs (shRNA #1, shRNA #2, and shRNA #3) or a non-targeting control shRNA, followed by immunoblotting with an anti-PAICS (A) or anti-α-tubulin (B) antibody.

Figure S8

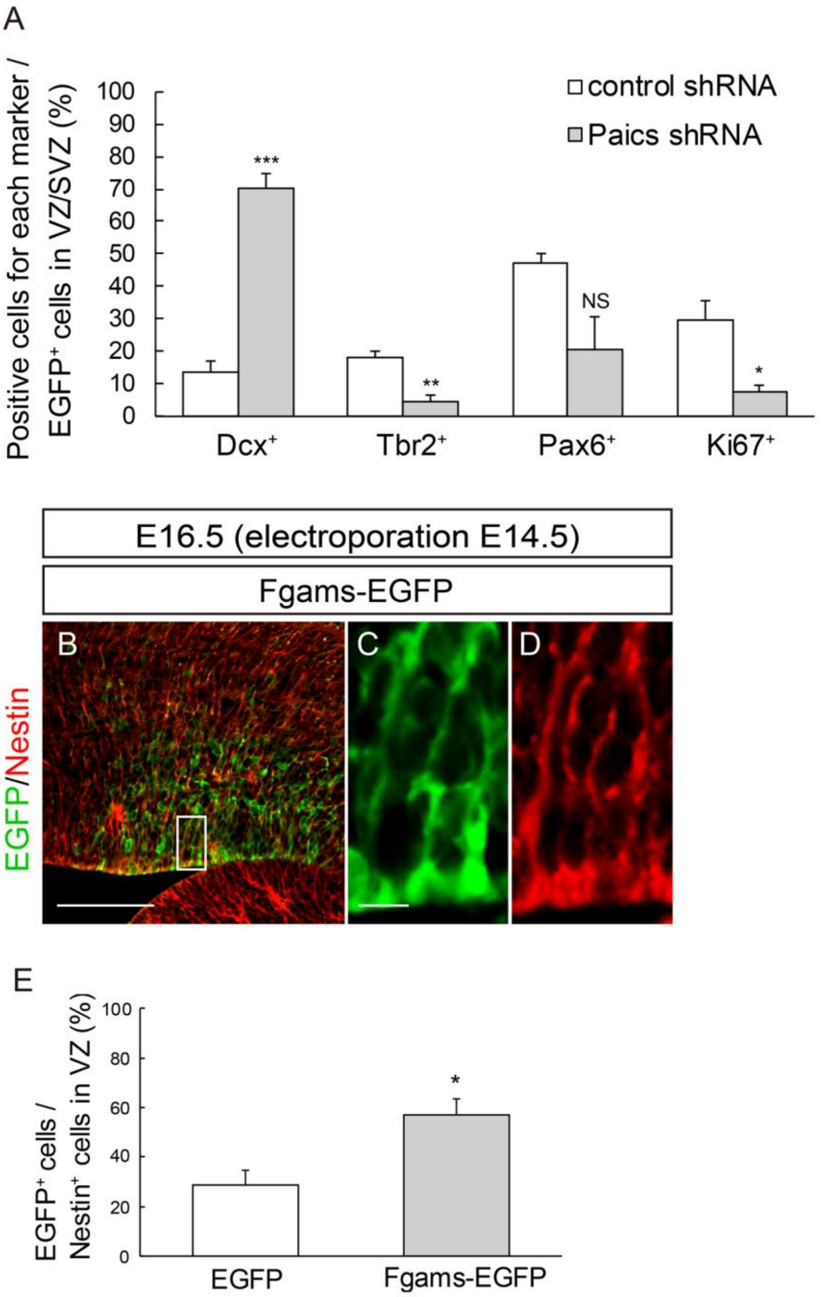
A
Positive cells for each marker / EGFP⁺ cells in VZ/SVZ (%)
100
90
80
70
60
50
40
30
20
10
0
control shRNA
Paics shRNA
***
**
NS
*
Dcx⁺
Tbr2⁺
Pax6⁺
Ki67⁺
E16.5 (electroporation E14.5)
Fgams-EGFP
B
C
D
EGFP/Nestin
E
EGFP⁺ cells / Nestin⁺ cells in VZ (%)
100
80
60
40
20
0
*
EGFP
Fgams-EGFP

Figure S8. Purinosome components regulate the cortical development. Related to Figure 8.

(A) Control shRNA or Paics shRNA was delivered into the brain on E14.5, together with EGFP, and the cortices were stained with anti-Dcx, anti-Pax6, anti-Tbr2, and anti-Ki67 antibodies at E16.5. Graph shows the quantification of the positive cells for each marker to total $EGFP^+$ cells in the VZ/SVZ. NS, not significant, *p<0.05, **p<0.01, ***p<0.001. Note that the knockdown of Paics gene accelerates the mitotic exit of $Pac6^+$ or $Tbr2^+$ NSPCs and induces the premature expression of neuron marker Dcx. (B–E) Fgams-EGFP was electroporated into the E14.5 neocortex and embryos were analyzed at E16.5. The cerebral cortex was stained with an anti-Nestin antibody (red). (C, D) Magnified view of the boxed area depicted in (B), showing that Fgams-$EGFP^+$ cells were persistently located in the VZ/SVZ as $Nestin^+$ NSPCs. (E) Quantification of Fgams-$EGFP^+$ cells to total $Nestin^+$ cells in VZ. *p<0.05. All data are presented as means ± SEM. Statistical significance value was determined using the Welch's *t*-test. Scale bars, 100 µm in B; 10 µm in C and D.

Table S1

Gene name	Gene Symbol	NCBI Reference Sequence: protein	Identified protein region (amino acids)	protein function
abhydrolase domain containing 3	Abhd3	NP_598891.1	350–411 aa	unknown, paralog of Abhd1
ATP-binding cassette, sub-family D (ALD), member 3	Abcd3	NP_033017.2	530–659 aa	peroxisomal import of fatty acids and/or fatty acyl-CoAs
chymotrypsin-like elastase family, member 1	Cela1	NP_291090.2	14–155 aa	protease associated with elastin remodeling
clavesin 2	Clvs2	NP_001346068.1	35–181 aa	recycling of synaptic vesicles
Kin17 DNA and RNA binding protein	Kin	NP_079556.1	1–188 aa	DNA/RNA binding protein
phosphoribosylaminoimidazole carboxylase, phosphoribosylaminoribosylaminoimidazole, succinocarboxamide synthetase	Paics	NP_080215.1	323–425 aa, 312–425 aa, 305–425 aa	*De novo* purine synthesis enzymes
quaking	Qk	NP_001152988.1	130–319 aa	RNA-binding protein
serine (or cysteine) peptidase inhibitor, clade E, member 2	Serpine2	NP_033281.1	307–397 aa	inhibititor for serine proteases
serine palmitoyltransferase, small subunit A	Sptssa	NP_598815.2	10–71 aa	serine palmitoyltransferase isoenzymes
E26 avian leukemia oncogene 1, 5' domain	Ets1	NP_001359463.1	1–111 aa	transcription factor
tripeptidyl peptidase II	Tpp2	NP_033444.1	1171–1261 aa	serine exopeptidase
WD repeat domain 74	Wdr74	NP_598900.1	114–378 aa	regulator of exosome complex formation

Table S1. Nwd1-binding partners identified by yeast two-hybrid screen. Related to Figure 5.

Each protein entry is shown with the respective NCBI accession number and the identified protein region. The respective protein functions are also listed.

Transparent Methods

Animals

ICR male mice were purchased from Japan SLC Inc. (Shizuoka, Japan). The date of conception was established by the presence of a vaginal plug and recorded as embryonic day zero (E0). The day of birth was designated as P0. Mice were housed under temperature- and humidity-controlled conditions on a 12/12 hr light/dark cycle, with *ad libitum* access to food and water. All protocols were approved by the Committee on the Ethics of Animal Experiments of Waseda University.

Tissue Preparation

Embryos at E16.5 and E18.5 were perfused through the cardiac ventricle with 4% paraformaldehyde (PFA) in 0.1 M phosphate buffer (pH 7.4), followed by post-fixation overnight at 4ºC (Yamada and Sakakibara, 2018). Fixed embryo brains were cryoprotected in 30% sucrose in phosphate-buffered saline (PBS) overnight at 4°C and embedded in optimal cutting temperature compound (Sakura Finetek). Frozen sections were cut at a thickness of 14 µm using a cryostat and were collected on MAS coated glass slides (Matsunami Glass).

Plasmid Vectors

Mouse *Nwd1* cDNAs were subcloned into the pEGFP-C2 vector (Clontech Takara Bio) to express the Nwd1 protein fused with EGFP. The *Nwd1* and *EGFP-Nwd1* cDNAs were subcloned into the pCAGGS vector (a gift from Dr. Jun-ichi Miyazaki, Osaka University, Japan). For the yeast two-hybrid (Y2H) screening, *Nwd1* cDNAs corresponding to the N-terminal portion of the protein (accession number BC082552; 4 bp–1026 bp) were

subcloned into pGBKT7 (Clontech Takara Bio) to express the N-terminal domain of Nwd1 fused with the GAL4 DNA-binding domain. The Flag-tagged human NWD1 expression plasmid was provided by Dr. Correa (Sanford-Burnham Medical Research Institute, Canada) (Correa et al., 2014). *pFGAMS-EGFP* and *pPAICS-EGFP* (# 99108) were gifts from Dr. Stephen Benkovic (Addgene plasmids # 99107 and # 99108, respectively) (An et al., 2008). HSP90-HA was a gift from Dr. William Sessa (Addgene plasmid #22487) (Garcia-Cardena et al., 1998). *pCAG-DsRED* was a gift from Dr. Connie Cepko (Addgene plasmid # 11151) (Matsuda and Cepko, 2004).

shRNA Expression Vectors

We purchased a MISSION shRNA vector library encoding the microRNA-adapted shRNA targeting mouse *Nwd1* (Sigma-Aldrich). Among five shRNA clones (TRCN0000257630, TRCN0000247062, TRCN0000257635, TRCN0000257616, and TRCN0000179877), TRCN0000247062 and TRCN0000257635 yielded efficient knockdown of the exogenous Nwd1 and EGFP-Nwd1 expressed in cultured cells; these clones were designated as shRNA #1 and shRNA #2, respectively. The targeting sequences of shRNA #1 and shRNA #2 were: 5′-TACGACTGTGCATGCTCTAAA-3′ and 5′-CAGGTAATCCAAGTTCGATAT-3′, respectively. The two constructs targeted the coding region of the Nwd1 mRNA. We also used MISSION shRNA plasmids for mouse *Paics* (Sigma-Aldrich). Among five clones (TRCN0000076100, TRCN0000076101, TRCN0000076102, TRCN0000076098, and TRCN0000076099), TRCN0000076101, TRCN0000076102, and TRCN0000076098 were designated as shRNA #1, shRNA #2, and shRNA #3, respectively. The targeting sequences for shRNA #1, shRNA #2, and shRNA #3 were 5′-CTGCTCAGATATTTGGGTTAA-3′, 5′-

GCTGATGTCATTGATAATGAT-3′, and 5′-GCACCTGCTTTCAAATACTAT-3′, respectively. shRNA #1 and shRNA #2 targeted the coding region, whereas shRNA #3 targeted the 3′ untranslated region (3′-UTR) of the *Paics* mRNA. A non-targeting shRNA (# SHC202) was also purchased from Sigma-Aldrich.

Primary Antibodies

The following primary antibodies were used: anti-Nwd1 (affinity-purified rabbit polyclonal antibody used previously (Yamada and Sakakibara, 2018), 1:200 for immunostaining, 1:2000 for immunoblotting), anti-Nwd1 (rabbit polyclonal antibody generated by immunizing the recombinant mouse Nwd1 protein; 1:500 for immunostaining, 1:5000 for immunoblotting), anti-Nestin (chicken polyclonal IgY, Aves Labs, NES; 1:4000 for immunostaining), anti-Nestin (rabbit polyclonal, IBL, 18741; 1:250), anti-α-tubulin (rabbit polyclonal, MBL, PM054; 1:2000 for immunoblotting), anti-GFP (chicken polyclonal IgY, Aves Labs, GFP-1010; 1:2000 for immunostaining), anti-GFP (rabbit polyclonal, GeneTex , GTX113617; 1:2000 for immunoblotting), anti-doublecortin (DCX) (goat polyclonal, Santa Cruz, sc-271390; 1:200 for immunostaining), anti-β-tubulin III (chicken polyclonal IgY, AVES Labs, TUJ; 1:1000 for immunostaining), anti-Pax6 (rabbit polyclonal, MBL, PD022; 1:1000 for immunostaining), anti-Tbr2 (chicken polyclonal, Merck Millipore, 633572; 1:1000 for immunostaining), anti-Ki67 (rabbit monoclonal clone SP6, Lab Vision, RM-9106; 1;1000 for immunostaining), anti-Paics (rabbit polyclonal, Proteintech, 12967-1-AP; 1:200 for immunostaining), anti-GFAP (mouse monoclonal clone G-A-5, Sigma-Aldrich, G3893; 1:400 for immunostaining), anti-SMI312 (mouse monoclonal, Biolegend, 837904; 1:1000, for immunostaining), anti-HA (rabbit polyclonal, MBL, 561; 1:200 for

immunostaining, 1:2000 for immunoblotting), and anti-DDDDK (Flag) (mouse monoclonal, MBL, FLA-1; 1:10000 for immunostaining and immunoblotting), anti-VGluT1 (rabbit polyclonal, GeneTex, GTX133148; 1:250 for immunostaining), anti-Tbr1 (rabbit polyclonal, GeneTex, GTX117615; 1:250 for immunostaining), anti-Brn2 (rabbit polyclonal, GeneTex, GTX114650; 1:250 for immunostaining), anti-MAP2 (chicken polyclonal IgY, Biolegend, 822591, 1:1000 for immunostaining), anti-BrdU (sheep polyclonal, Abcam, 1:1500 for immunostaining).

Cell Culture

HEK293T, HeLa, and Neuro2a (N2a) cells were cultured in Dulbecco's Modified Eagle Medium (DMEM) containing 10% fetal bovine serum (FBS), penicillin/streptomycin, and L-glutamine. In purine-depleted conditions, HeLa cells were cultured in RPMI 1640 medium supplemented with L-glutamine and 5% dialyzed FBS. FBS was dialyzed against 0.9% NaCl using a 10K MWCO SnakeSkin Tubing (Thermo Fisher Scientific) for 48 hr at 4°C (An et al., 2008; French et al., 2013). NSPCs were isolated from the E12.5 telencephalon, seeded onto dishes coated with fibronectin and polyethylenimine (PEI) (Sigma-Aldrich), and cultured in Advanced DMEM/F-12 (1:1) (Life Technologies) supplemented with 15 μg/mL insulin (Life Technologies), 25 μg/mL transferrin (Life Technologies), 20 nM progesterone (Sigma-Aldrich), 30 nM sodium selenite (Sigma-Aldrich), 60 nM putrescine (Sigma-Aldrich), 20 ng/mL basic fibroblast growth factor (FGF2) (Merck Millipore), and 10 ng/mL epidermal growth factor (Merck Millipore) (Yamada and Sakakibara, 2018; Yumoto et al., 2013). To assess cell proliferation, 10 μM BrdU (5-bromodeoxyuridine, Tokyo Chemical Industry) was administrated to the primary cultured NSPCs. After 24 hr of incubation, cells were cultivated in the medium

without BrdU for the indicated periods, and then processed for immunostaining using anti-BrdU antibody. For the differentiation assay of NSPCs, culture medium was changed from the proliferation medium to the EGF and FGF2-free differentiation medium containing 1% FBS. For the primary culture of cortical neurons, embryonic cerebral cortices at E16.5 were dissected and mechanically dissociated. After washing with Opti-MEM I (Life Technologies), cells were electroporated, seeded onto poly-D-lysine-coated dishes, and cultured in neurobasal medium containing 2% B27 (Life Technologies) and 1% GlutaMax (Life Technologies) for 1–3 days *in vitro* (div). For immunostaining, cultured cells were fixed with 4% PFA for 20 min at 4℃ and permeabilized in 0.05% Triton X-100 in PBS for 10 min.

Cell Transfection

Cultured cell lines were transfected with plasmid DNA and PEI MAX (Polysciences) complexes (ratio of DNA to PEI MAX, 1:3, w/w) formed in Opti-MEM I by incubation for 15 min at room temperature. The DNA complexes were added to cell cultures together with Opti-MEM I for 3 hr, followed by cultivation with serum containing complete DMEM. Mouse NSPCs were expanded *in vitro* as described above, and primary cortical neurons were electroporated using a NEPA21 Electroporator (Nepagene) according to the manufacturer's specifications (NSPCs: two pulses of 125 V for 5 ms with an interval of 50 ms; primary cortical neurons, two pulses of 275 V for 0.5 ms with an interval of 50 ms).

In utero Electroporation

Pregnant mice were anesthetized *via* intraperitoneal injection of a mixture containing medetomidine, midazolam, and butorphanol (Kawai et al., 2011). A DNA solution (5 μg/μL) in PBS with 0.01% Fast Green dye (Sigma-Aldrich) was injected into the lateral ventricle through the uterus wall, followed by electroporation (Yumoto et al., 2013). The following constructs were electroporated: *pCAG-Nwd1*, *pCAG-EGFP*, *pCAG-EGFP-Nwd1*, *pFgams-EGFP*, *Nwd1* shRNAs, *Paics* shRNAs, and non-targeting shRNA. Electric pulses were generated by NEPA21 (Nepagene) and applied to the cerebral wall using a platinum oval electrode (CUY650P5, Nepagene), with four pulses of 35 V for 50 ms with an interval of 950 ms. An anionic electrode was placed on the lateral cortex, to ensure the incorporation of DNA into the VZ/SVZ. Embryos were perfused at E16.5, E18.5, and P7 with 4% PFA through cardiac perfusion.

Immunostaining

Frozen sections were blocked for 2 hr with 5% normal goat or donkey serum in PBST (0.1% Triton X-100 in PBS), followed by the incubation with primary antibodies in blocking buffer at 4°C overnight (Yamada and Sakakibara, 2018). After washing with PBST four times, sections were incubated for 2 hr with Alexa Fluor 488-, Alexa Fluor 555- (Life Technologies), or DyLight 488-, DyLight 549- (Jackson ImmunoResearch) conjugated secondary antibodies. After counterstaining with 0.7 nM Hoechst 33342 (Life Technologies), sections were mounted and imaged using a confocal (FV3000, Olympus) or fluorescence inverted microscope (Axio Observer, Zeiss). For BrdU staining, fixed cells were treated with 2 N HCl for 1 hr at 37°C to denature the DNA, followed by the treatment with 0.1 M sodium borate buffer (pH 8.5) for 10 min (Sakakibara et al., 2001). After washing with PBS, anti-BrdU immunostaining was performed.

Immunoprecipitation and Western Blotting

For immunoprecipitation (IP), cells were washed in ice-cold PBS and lysed in ice-cold lysis buffer containing 50 mM Tris-HCl (pH 7.5), 150 mM NaCl, 2 mM EDTA, 1% NP-40, and protease inhibitors (cOmplete Mini Protease Inhibitor Cocktail, Roche Diagnostics) for 30 min at 4ºC. Lysates were centrifuged at 15,000 rpm for 10 min and the supernatants were precleared with TrueBlot anti-rabbit or anti-mouse IP beads (Rockland Immunochemicals) for 1 hr. After centrifugation at 15,000 rpm for 10 min, the supernatants were incubated with the primary antibody coupled to TrueBlot IP beads (Rockland Immunochemicals) overnight or 1 hr at 4°C. Rabbit or mouse IgG (Thermo Fisher Scientific) was used as a control. After brief centrifugation, beads were washed four times with 0.1% NP40 in PBS, and then the bound proteins were dissolved by treatment with 2×sample buffer (125 mM Tris-HCl, pH 6.8, 4% SDS, 10% sucrose, and 0.01% bromophenol blue). IP samples were resolved on 8% or 10% SDS-PAGE, and electroblotted onto Immobilon-P membranes (Merck Millipore) using a semidry transfer apparatus. After blocking with 5% skim milk in TBST (150 mM NaCl, 10 mM Tris−HCl, pH 7.4, 0.1% Tween 20), membranes were incubated with primary antibody for 1 hr, followed by incubation with the horseradish peroxidase (HRP)-conjugated secondary antibody (GE Healthcare). The signal was detected using the Immobilon western chemiluminescent HRP substrate (Merck Millipore) and visualized by Fusion Solo S (Vilber Lourmat).

TUNEL staining

TUNEL (Terminal deoxynucleotidyl transferase dUTP nick end labeling) assay was performed for detecting apoptosis using the In Situ Cell Death Detection Kit, TMR-red (Roche Diagnostics), according to the manufacturer's instruction.

Yeast Two-hybrid Screening

To identify Nwd1-binding proteins in the CNS, a Y2H screening was performed using the Matchmaker Gold Yeast Two-Hybrid System (Clontech Takara Bio), according to the manufacturer's instructions. The sequence encoding the 340 N-terminal amino acid residues of mouse Nwd1 (BC082552; 4–1026 bp) was subcloned in frame into pGBKT7, to express the N-terminal region of Nwd1 (bait) fused with the GAL4 DNA-binding domain. As the prey, we used the normalized mouse brain library (Clontech Takara Bio, Normalized Mate & Plate Library, cat. # 630488), in which each clone was fused with the Gal4 DNA-activating domain (AD). Mated yeast clones were selected using minimal synthetic defined (SD) medium with double dropout (Leu^{-} and Trp^{-}) supplement (Clontech Takara Bio) containing Aureobasidin A and X-α-Gal as the blue-colored colonies; this was followed by a second screening using the SD quadruple dropout (Leu^{-}, Trp^{-}, Ade^{-}, and His^{-}) selective medium (Clontech Takara Bio). After the elimination of duplicates containing the same AD/library plasmid via yeast-colony PCR, plasmids were rescued from yeast using the Easy Yeast Plasmid Isolation Kit (Clontech Takara Bio). Protein interactions were confirmed by co-transformation of the Nwd1 bait with each candidate prey plasmid into Y2H Gold yeast host cells, followed by sequencing of the cDNA inserts.

Statistical Analyses

All numerical data are expressed as means ± SEM. In two-group comparisons, Welch's *t*-test was used to assess the significance of the differences in cell distribution over the cortical layers, positive cells for each marker, the number of neurites, or the number of purinosomes between different groups. In multiple-group comparisons, analysis of variance followed by Welch's *t*-test was used. The *p* values obtained were corrected for multiple testing using the Holm–Bonferroni correction. The chi-squared test was used for comparison with the number of $BrdU^{+}$ or β-tubulin III^{+} cells in the NSPCs proliferation/differentiation assays, and the number of $SMI312^{+}$ axons counted in the neuronal differentiation assay.

Supplemental References

Garcia-Cardena, G., Fan, R., Shah, V., Sorrentino, R., Cirino, G., Papapetropoulos, A., and Sessa, W.C. (1998). Dynamic activation of endothelial nitric oxide synthase by Hsp90. Nature *392*, 821–824.

Kawai, S., Takagi, Y., Kaneko, S., and Kurosawa, T. (2011). Effect of three types of mixed anesthetic agents alternate to ketamine in mice. Exp. Anim. *60*, 481–487.

Matsuda, T., and Cepko, C.L. (2004). Electroporation and RNA interference in the rodent retina in vivo and in vitro. Proc. Natl. Acad. Sci. USA *101*, 16–22.

Sakakibara, S., Nakamura, Y., Satoh, H., and Okano, H. (2001). RNA-binding protein Musashi2: developmentally regulated expression in neural precursor cells and subpopulations of neurons in mammalian CNS. J. Neurosci. *21*, 8091–8107.

Yumoto, T., Nakadate, K., Nakamura, Y., Sugitani, Y., Sugitani-Yoshida, R., Ueda, S., and Sakakibara, S. (2013). Radmis, a novel mitotic spindle protein that functions in cell division of neural progenitors. PLoS One *8*, e79895.
